# Spinal motoneuron firing properties mature from rostral to caudal during postnatal development of the mouse

**DOI:** 10.1113/JP280274

**Published:** 2020-09-16

**Authors:** Calvin C. Smith, Robert M. Brownstone

**Affiliations:** ^1^ Department of Neuromuscular Diseases UCL Queen Square Institute of Neurology University College London London UK

**Keywords:** critical period, electrophysiology, locomotion, motor development, movement, spinal cord

## Abstract

**Key points:**

- Many mammals are born with immature motor systems that develop through a critical period of postnatal development.
- In rodents, postnatal maturation of movement occurs from rostral to caudal, correlating with maturation of descending supraspinal and local spinal circuits.
- We asked whether development of fundamental electrophysiological properties of spinal motoneurons follows the same rostro‐caudal sequence.
- We show that in both regions, repetitive firing parameters increase and excitability decreases with development; however, these characteristics mature earlier in cervical motoneurons.
- We suggest that in addition to autonomous mechanisms, motoneuron development depends on activity resulting from their circuit milieu.

**Abstract:**

Altricial mammals are born with immature nervous systems comprised of circuits that do not yet have the neuronal properties and connectivity required to produce future behaviours. During the critical period of postnatal development, neuronal properties are tuned to participate in functional circuits. In rodents, cervical motoneurons are born prior to lumbar motoneurons, and spinal cord development follows a sequential rostro‐caudal pattern. Here we asked whether birth order is reflected in the postnatal development of electrophysiological properties. We show that motoneurons of both regions have similar properties at birth and follow the same developmental profile, with maximal firing increasing and excitability decreasing into the third postnatal week. However, these maturative processes occur in cervical motoneurons prior to lumbar motoneurons, correlating with the maturation of premotor descending and local spinal systems. These results suggest that motoneuron properties do not mature by cell autonomous mechanisms alone, but also depend on developing premotor circuits.

## Key points

- Many mammals are born with immature motor systems that develop through a critical period of postnatal development.
- In rodents, postnatal maturation of movement occurs from rostral to caudal, correlating with maturation of descending supraspinal and local spinal circuits.
- We asked whether development of fundamental electrophysiological properties of spinal motoneurons follows the same rostro‐caudal sequence.
- We show that in both regions, repetitive firing parameters increase and excitability decreases with development; however, these characteristics mature earlier in cervical motoneurons.
- We suggest that in addition to autonomous mechanisms, motoneuron development depends on activity resulting from their circuit milieu.

## Introduction

Altricial mammals are born with immature nervous systems comprised of circuits that have neither the neuronal properties nor connectivity required to produce future behaviours. During the critical period of postnatal development, neuronal properties are tuned to participate in functional circuits. The degree to which these mature electrophysiological properties are determined by cell‐intrinsic *vs*. extrinsic (e.g. circuit) factors is not clear. To understand how neural circuits are ultimately tuned requires an understanding of how properties of their component neurons develop.

A model system in which to understand this is the spinal cord, where two distinct regions – the cervical and the lumbar cord – include homologous circuits, with both regions containing circuitry to coordinate the intra‐ and inter‐limb movements required for locomotion (Jessell, 2000a; Goulding, 2009). The output from each region is mediated by motoneurons, which have overlapping molecular profiles in the two regions. During embryonic development, cervical motoneurons are born a few days before lumbar motoneurons in mice, rats and chicks (Nornes & Das, 1974; Hollyday & Hamburger, 1977), and these regions develop sequentially from rostral to caudal (Sagner & Briscoe, 2019). Is this developmental trajectory maintained in the postnatal critical period such that the electrophysiological properties of cervical motoneurons are more mature at birth and then fully mature before lumbar motoneurons? Or do the development and maturation of these properties rely on the development of circuits, such as descending and sensory inputs; in which case the two populations would be born with similar properties that mature as movement develops during the postnatal critical period?

There is little question that both behaviour and motoneuron properties develop postnatally. Rodents are essentially immobile in the first 2 days following birth, until forelimb‐propelled pivoting and crawling become the dominant forms of ambulation during the first week of life (Altman & Sudarshan, 1975; Sechzer *et al*. 1984). Quadrupedal locomotion subsequently emerges around P10–12 when hindlimbs consistently support the weight of the lower quadrant (Jiang *et al*. 1999), with locomotor maturity reached by the end of the third postnatal week (Altman & Sudarshan, 1975). Lumbar motoneuron properties change at least in the first postnatal week, the period (and region) in which electrophysiological experiments are usually done (Fulton & Walton, 1986; Nakanishi & Whelan, 2010; Quinlan *et al*. 2011). But do these changes in properties proceed from rostral to caudal?

Many aspects of spinal circuit development ensue from rostral to caudal. Descending supraspinal pathways arrive and mature in cervical segments earlier than in lumbar segments. In rodents, fibres originating in the brainstem are the first to arrive in the spinal cord, sprouting into the grey matter of the cervical cord prenatally, and reaching the lumbar spinal segments at or shortly after birth (Vinay *et al*. 2000). Descending modulatory systems follow a similar pattern: serotonergic innervation of the cervical spinal cord displays the adult profile by P14, whereas the fibre density in the lumbar cord does not mature until P21 (Bregman, 1987). Similarly, corticofugal axons innervate the cervical grey matter at P5–6 and arrive at the lumbar cord at approximately P9. But it is not until after P21 that all segments display the mature density and pattern of descending innervation (Donatelle, 1977; Gianino *et al*. 1999). Sensory innervation of the spinal cord follows a similar trend, as cutaneous reflexes can be evoked in the muscles of the neck and forelimbs (E16–17) before those in the hindlimbs (E17–18) (Narayanan *et al*. 1971). Furthermore, postnatal refinement of proprioceptive afferent input to motoneurons is dependent upon the maturation of descending input to the spinal cord and does not mature until the end of the third postnatal week in rats (Smith *et al*. 2017). Thus, key spinal circuit development occurs in the first three postnatal weeks and, where studied, proceeds from the cervical to the lumbar spinal cord.

Do motoneuron properties follow this same pattern? In addition to being born earlier, anatomical data indicate that cervical motoneurons reach adult motoneuron size before the lumbar motoneurons (Cameron *et al*. 1989). In rats, the appearance of spontaneous burst activity in embryonic cervical motoneurons precedes that in lumbar motoneurons by about one embryonic day (Nakayama *et al*. 1999). Importantly, it is clear that the transcriptional profile rather than circuit milieu (and limb movement) is sufficient for basic motoneuron properties to develop: motoneurons derived in culture from embryonic stem cells develop electrophysiological properties characteristic of spinal motoneurons (Miles *et al*. 2004).

We thus reasoned that, not only would motoneuron properties develop in the early postnatal period, but that if transcriptional profile alone were responsible for this maturation, then cervical motoneurons would be more mature at the time of birth and reach adult‐like properties earlier than lumbar motoneurons. On the other hand, if circuit properties and limb movement are critical for maturation, then cervical and lumbar motoneurons would be born with similar properties that then mature postnatally. To study this question, we used whole‐cell patch clamp recordings of mouse cervical and lumbar motoneurons to define and compare their electrophysiological properties at three time points during this critical period of development from birth to weaning (P2–3, P6–7 and P14–21). We demonstrate that the two populations are born with similar properties and then develop through the third postnatal week. Furthermore, cervical motoneuron properties mature earlier than those of lumbar motoneurons. We thus suggest that transcriptional profiles alone are insufficient for this maturation, and that the postnatal development of circuits contributes to the tuning of neuronal properties.

## Methods

### Confirmation of compliance

The authors confirm that they understand, and that this work complies with, the ethical principles under which the *Journal of Physiology* operates (Grundy, 2015).

### Ethical approval

Experiments were approved by the University College London Animal Welfare and Ethical Review Body and performed under a licence granted under the Home Office Animals (Scientific Procedures) Act 1986.

### Animals

All experiments were carried out on Hb9::eGFP mice of both sexes aged P2–P21. Male Hb9::eGFP (B6.Cg‐Tg(Hlxb9‐GFP)1Tmj/J) mice were acquired initially from Tom Jessell's lab and have been bred in the Brownstone lab since the early 2000s. This strain is available from JAX (stock no. 005029, RRID:IMSR_JAX:005029). We bred them with C57BL/6 mouse wild‐type females acquired from Charles River Laboratories, Inc (strain code:027). The date of birth was called P0 and recordings were made at three different age ranges (P2–3, P6–7 and P14–21). A total of 54 animals were used in the study (lumbar P2–3, *n* = 7; cervical P2–3, *n* = 6; lumbar P6–7, *n* = 11; cervical P6–7, *n* = 11; lumbar P14–21, *n* = 8, cervical P14–21, *n* = 11). For all measures except afterpotentials at P6–7 (lumbar: *n* = 20, cervical: *n* = 19), cell numbers at each age for each region were as follows: lumbar and cervical P2–3, *n* = 17; lumbar and cervical P6–7, *n* = 30; lumbar P14–21, *n* = 18, cervical P14–21, *n* = 22. The average age of mice from which P14–21 lumbar motoneurons were sampled was 17.1 ± 2.0 days and 16.2 ± 1.7 days for cervical motoneurons. Motoneurons from lumbar or cervical segments were always sampled from separate mice.

### Spinal cord isolation

Lumbar spinal cords: Animals were deeply anaesthetized by intraperitoneal injection of a ketamine (100 mg kg^‐1^) and xylazine (20 mg kg^‐1^) mixture. Upon loss of paw withdrawal, mice were then decapitated and the vertebral column was quickly isolated and pinned down (ventral side up) in a dissecting dish containing ice‐cold (0–4°C) normal artificial cerebrospinal fluid (nACSF) saturated with 95% carbogen. The composition of the nACSF was as follows (in mm): 113 NaCl, 3 KCL, 25 NaHCO_3_, 1NaH_2_PO_4_, 2 CaCl, 2 mgCl_2_ and 11 D‐glucose, pH 7.4 (Mitra & Brownstone, 2012). The vertebral bodies were removed to reveal the spinal cord, the roots were cut and dura matter removed. The spinal cord was isolated from rostral thoracic to caudal sacral levels, placed upon tissue paper to soak up excess liquid and then the dorsal side glued (3M Vetbond, No.1469SB) to a pre‐cut block of agarose mounted on a cutting chuck.

Cervical spinal cords: For cervical slices (also under deep anaesthesia), a craniotomy was done to expose and remove the cerebellum, and the brainstem was transected at the level of the obex. All nervous tissue rostral to the transection was immediately removed. This process was critical for preserving cervical spinal tissue. The vertebral column was then transferred to a dissecting dish (as with the lumbar preparation), pinned dorsal side up and a laminectomy performed to a mid‐thoracic level. Roots were cut, the dura removed and cervical cord glued to the chuck in the same fashion as for the lumbar cord.

### Slice preparation

The cord was transferred to a vibrating microtome (Model 7000smz‐2, Campden Instruments Ltd) chamber containing ice‐cold slicing solution made up of the following (in mm): 130 potassium gluconate, 15 KCL, 0.05 EGTA, 20 HEPES, 25 glucose, 3 kynurenic acid, pH 7.4 (Dugué *et al*. 2005; Bhumbra & Beato, 2018). Slices were made at 350 μm, transferred to a recovery chamber containing nACSF (32°C) for 30 min and then left to equilibrate to room temperature for at least 30 min before recording (1 h total post‐slice recovery).

### Electrophysiology

Motoneuron recordings were made with a MultiClamp 700A amplifier (Axon Instruments, Inc), low pass filtered at 10 kHz and digitized at 25 kHz using a CED Power3 1401 and Signal software (Cambridge Electronic Design Ltd, Cambridge, UK, RRID:SCR_01 7282). Patch pipette electrodes were pulled with a horizontal puller (P‐97 Flaming/Brown Micropipette Puller; Sutter Instrument, RRID:SCR_01 6842) to a resistance of 1–5 mΩ and filled with the internal recording solution made up of (in mM): 131 K‐methanesulfonate, 6 NaCl, 0.1 CaCl_2,_ 1.1 EGTA, 10 HEPES, 0.3 mgCl_2_, 3 ATP‐Mg, 0.5 GTP‐Na, 2.5 L glutathionine, 5 phosphocreatine, pH 7.25 adjusted with KOH, osmolarity 290–300 mOsm.

Large eGFP‐positive neurons in the ventral lumbar and cervical spinal cord were identified as motoneurons (Wilson *et al*. 2005). Motoneurons were patched at room temperature (approx. 21°C) using infrared‐differential interference contrast (IR‐DIC) optics on a DMLFSA microscope (Leica DMLFSA; Leica Microsystems, Wetzlar, Germany). All lumbar motoneurons were selected from the lateral motor nuclei of segments L1–6 and all cervical cells were selected from the lateral motor nuclei of slices made from C4–8. It is therefore likely that the majority (if not all) of cells included in our study were limb‐innervating motoneurons (Watson *et al*. 2009; Mohan *et al*. 2014; Mohan *et al*. 2015).

### Data analysis

All data were captured and analysed with CED Signal software (RRID:SCR_01 7081). Motoneurons were only analysed if they had a resting membrane potential (RMP) more hyperpolarized than ‐60 mV that did not deviate by more than 5 mV during recording.

All experiments were performed in current clamp mode. The bridge was balanced and capacitance neutralized prior to commencing recording. Motoneurons were injected with a small negative rectangular pulse (500 ms duration) and the voltage response of 15–30 traces was averaged to measure input resistance and whole‐cell capacitance (WCC). Resistance was measured as the peak voltage change to the injected current and τ calculated from an exponential curve fitted to the response (automated in Signal). WCC was calculated using resistance and τ values and cross‐checked against the values automatically recorded by the software during the experiment. I_min_ was defined as the minimum amount of current needed to evoke ≥ 2 action potentials (APs). This was tested with 500 ms duration, rectangular current pulses of increasing magnitude (0.03 nA steps) starting at ‐0.3 nA. Sag potentials were recorded by injecting 0.03 nA hyperpolarizing current steps (500 ms) from 0 to ‐1 nA. The sag amplitude was measured as the difference between the peak of the negative voltage deflection at the start of the 500 ms pulse and the steady state at the end. Motoneuron f‐I graphs were generated by injecting depolarizing current steps increasing from 0 nA until maximum firing was observed. The excitability of the cell (gain) was determined by measuring the slope of the linear portion of f‐I plots for spike number, initial frequency (first two spikes instantaneous frequency), and final frequency (steady state, final two spikes instantaneous frequency).

Action potential half widths (AP HWs), spike amplitude, maximum rate of depolarization/repolarization and fast afterhyperpolarization (fAHP) were measured from 15–30 averaged single APs evoked with a 20 ms rectangular current pulse. The AP HW was calculated as the time between the 50% rise and 50% fall in amplitude of the AP. Spike height was measured as the voltage difference between the threshold (voltage at maximum positive value of the second derivative of membrane potential of AP) and the peak of the AP. The fAHP was measured as the difference between the voltage baseline and the most negative point on the first trough of the AP. Phase–plane plots of the single APs were generated to calculate maximum rates of depolarization and repolarization. Afterpotential measurements (mAHP, mAHP half decay time and afterdepolarization (ADP) amplitude) were taken from single APs evoked with a 1 ms duration current pulse. The mAHP amplitude was calculated from baseline (held at ‐65 mV) to the most negative point on the trough. The mAHP half decay time is calculated as half the time taken (ms) from the most negative point of the mAHP to baseline. ADP amplitude was measured from the fAHP peak to the most positive value before the start of the mAHP repolarization. All cells were held at ‐65 mV for single AP experiments.

### Statistics

Data from all cells were initially exported to a Microsoft Excel file. All subsequent data processing and analysis was done using Python (RRID:SCR_0 08394) running through the Jupyter notebooks (RRID:SCR_01 8413) environment. All data processing, graphing and statistical procedures can be found, edited and re‐run in the ‘myBinder’ link below (see *Data deposition* section). Given the diversity of motoneurons in the spinal cord and in line with the three Rs for animal research, measures from individual cells were treated as the experimental unit (N).

There were three questions we wanted to ask of our data, which advised our statistical procedures. They were:

1. Is there sustained change in an electrophysiological parameter over development?
2. If there is a change, is that change localized to a particular time point?
3. If it is localized, when does that change occur?

To answer these questions we took the following approach:

1. Most of our parameters were expected to change monophasically throughout development. We therefore decided to use Spearman's correlation coefficient to determine the effect of age within each nominal class (lumbar and cervical). For sag slope, which showed a V shape development profile, we used a two‐way ANOVA with the two independent variables being age and region and the dependent variable sag slope.
2–3. Subsequently, in order to determine if the change was localized to a particular time point (2) and when that is (3), we performed corrected multiple pairwise comparisons using either Student's t tests (Gaussian distribution) or Mann–Whitney U tests (non‐Gaussian distribution). Distribution was assessed using the Shapiro–Wilk test. The Holm–Sidak method was used to correct P values for multiple comparisons. Following two‐way ANOVAs we used the Tukey method for pairwise comparisons.

All data are reported as means ± standard deviations. Graphs were produced using Seaborn and Python open‐source software. Split violin plots with individual observations (grey lines) and means (red) were used to show and compare the distribution of the data. Joint‐plots are based on the means of the data and were included to give a clear comparison of the developmental profile between cervical and lumbar regions. Final figures were produced using CorelDraw Home & Student X8 software (RRID:SCR_01 4235).

### Principal component analysis

Principal component analysis was performed in python using the Scikit‐learn package (Pedregosa *et al*. 2011). All data were scaled and fit‐transformed before analysis (see myBinder link below for full code).

## Results

To effect movement, motoneurons must have the machinery to integrate synaptic inputs and convert them to trains of APs of appropriate frequencies for the intended behaviour. The ability to do this processing arises from a combination of passive, transition and repetitive firing properties. Here, we report our findings on postnatal development in each of these categories, comparing limb‐innervating motoneurons within the cervical and lumbar spinal cord. For each measure (except sag slope) we first report whether there is a sustained change with age for cervical and lumbar motoneurons based on the results of a Spearman's rank test. If a significant correlation is found, we proceed to test for normality (Shapiro–Wilk) and then perform corrected pairwise comparisons using *t* tests or Mann–Whitney U tests between six groups (lumbar and cervical, P2–3, P6–7 and P14–21).

### Postnatal development of motoneuron passive properties

**Figure 1:**
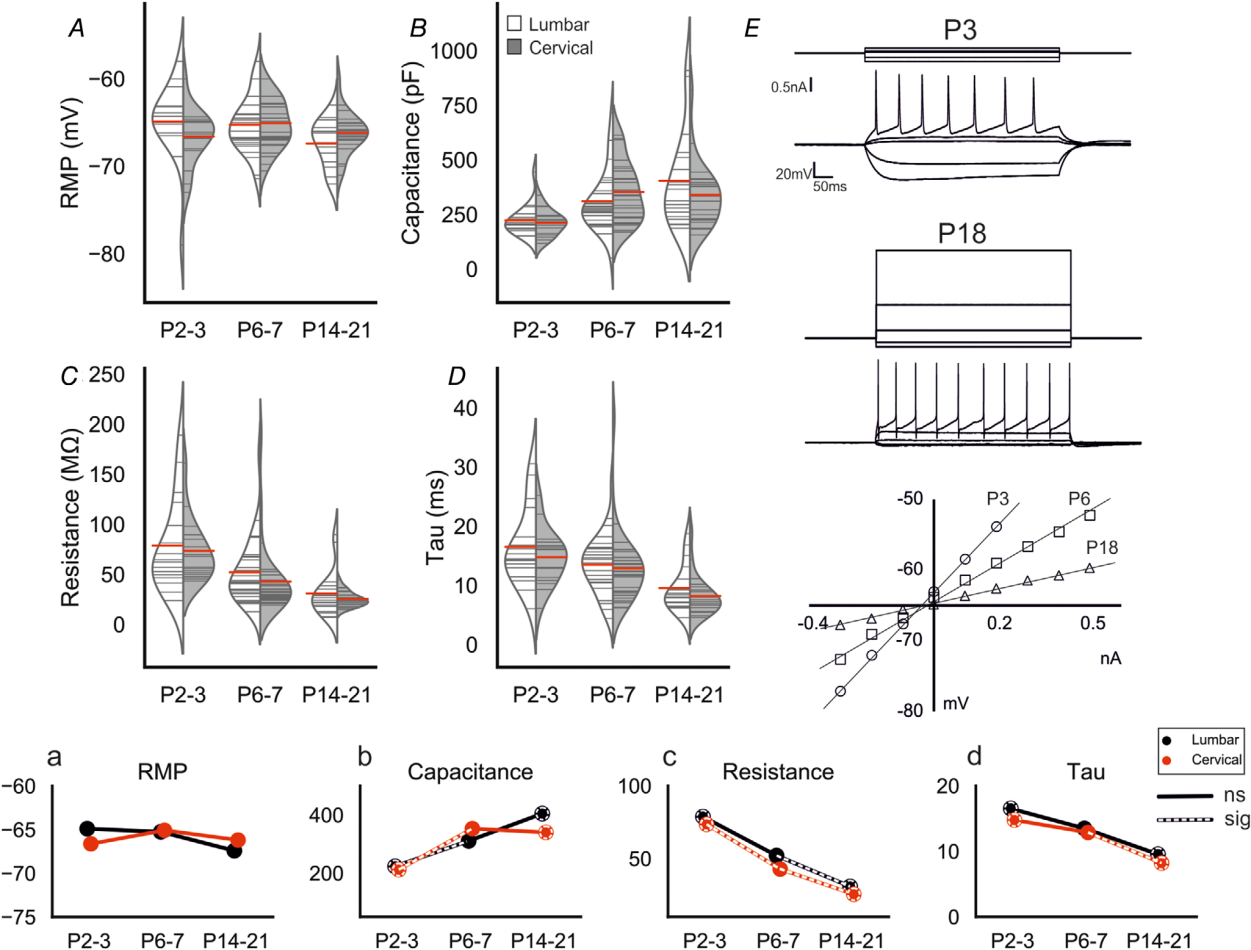
Postnatal development of motoneuron passive properties *A–D*, split violin plots showing development of passive membrane properties (lumbar‐white and cervical‐grey). Grey horizontal lines within violins show individual observations for each age and region. The red horizontal lines represent mean values. *E*, membrane voltage responses to 500 ms current pulses in a P3 and P18 motoneuron. For both cells the top trace is the injected current and the bottom trace the membrane voltage response. Lower: A graph showing peak membrane voltage response (*y*‐axis) to current input (*x*‐axis) for a representative motoneuron within each age group. (*a*–*d*) Joint‐plots of means illustrating the developmental profile of each measure in *A*–*D*. Overlaid white dashed lines represent statistically significant changes between age groups. Dashed circles represent statistically significant difference between P2–3 and P14–21. * represents a statistically significant difference (*p* < 0.05) between regions for a particular age group. Mann–Whitney U tests with Holm–Sidak corrected *P* values used for pairwise comparisons. Cell numbers: lumbar & cervical P2–3, *n* = 17; lumbar & cervical P6–7, *n* = 30; lumbar P14–21, *n* = 18, cervical P14–21, *n* = 22. [Color figure can be viewed at wileyonlinelibrary.com]

The key passive properties to consider are RMP, WCC, and input resistance. In lumbar motoneurons, there was a slight hyperpolarization in RMP with age (Spearman's ρ = ‐0.420, *p* = 0.001); however, there was no change in cervical motoneurons (Spearman's ρ= ‐0.074, *p* = 0.598). Comparisons between age groups in lumbar motoneurons revealed there were no significant differences (P2–3 = ‐64 *vs*. P6–7 = ‐65, *p* = 0.89; P6–7 *vs*. P14–21 = ‐67, *p* = 0.32; P2–3 *vs*. P14–21: *p* = 0.079), suggesting that RMP remains relatively constant during development in both regions (Fig. 1 *A, a*).

WCC reflects neuronal size, which for motoneurons has implications on behavioural output, including muscle fibre types innervated and recruitment order. There was an increase in WCC in both lumbar (Spearman's ρ = 0.502, *p* = 0.0001) and cervical motoneurons (Spearman's ρ = 0.343, *p* = 0.011, Fig. 1 *B, b*). In lumbar motoneurons, WCC increased in the first postnatal week (P2–3 = 223 ± 71 pF *vs*. P6–7 = 311 ± 140 pF, *p* = 0.036), but stabilized thereafter (P14–21 = 404 ± 217 pF, *p* = 0.71). A similar profile was seen for WCC in cervical motoneurons (P2–3 = 212 ± 59 pF *vs*. P6–7 = 353 ± 136 pF, *p* = 0.004; P6–7 *vs*. P14–21 = 340 ± 148 pF, *p* = 0.94). Together, these data suggest that both cervical and lumbar motoneurons increase in size postnatally at a similar rate.

Measurements of input resistance reflect the conductance properties of channels that open near resting potential, and also correlate with neuronal size. There was a significant effect of age on resistance for both lumbar (Spearman's ρ = ‐0.551, *p* = 1e‐05) and cervical (Spearman's ρ = ‐0.812, *p* = 2e‐13, Fig. 1 *C, c, E*) motoneurons. In lumbar motoneurons, there was no change between P2–3 (79 ± 47 mΩ) and P6–7 (52 ± 32 mΩ, *p* = 0.130), but there was a reduction thereafter (P6–7 *vs*. P14–21 = 31 ± 23 mΩ, *p* = 0.027). In cervical motoneurons resistance decreased earlier than in lumbar motoneurons as there were significant differences between all age groups (P2–3, 74 ± 31 mΩ *vs*. P6–7, 43 ± 34 mΩ, *p* = 2e‐04 and P6–7 *vs*. P14–21, 26 ± 8 mΩ, *p* = 0.011). There were no differences between lumbar and cervical motoneurons at any age (P2–3, *p* = 0.958; P6–7, *p* = 0.18; P14–21, *p* = 0.958). This indicates postnatal maturation of these conductances in both groups at similar rates.

Neuronal time constant, τ, combines WCC and input resistance, and reflects integrative properties of the cell. Analysis of τ in lumbar and cervical motoneurons revealed a developmental pattern similar to that seen for input resistance, as both decreased significantly with maturity (lumbar: Spearman's ρ = ‐0.407, *p* = 0.002; cervical: Spearman's ρ = ‐0.407, *p* = 6e‐08, Fig. 1 *D, d*). For lumbar motoneurons, this result reflects a gradual decrease in τ as it did not change between P2–3 (16 ± 6 ms) and P6–7 (13 ± 4 ms, *p* = 0.662) or P6–7 and P14–21 (9 ± 3 ms, *p* = 0.069), but there was a significant decrease between P2–3 and P14–21 (*p* = 0.015). In cervical motoneurons, τ decreased earlier than in lumbar motoneurons, between P6–7 and P14–21 (P6–7 = 12 ± 5 ms *vs*. P14–21 = 8 ± 3 ms, *p* = 0.001), but not P2–3 (14 ± 4 ms) and P6–7 (8 ± 3 ms, *p* = 0.329). Together, these data indicate that the overall, passive properties of motoneurons develop similarly in lumbar and cervical motoneurons.

### Postnatal development of motoneuron transition properties: action potentials

**Figure 2:**
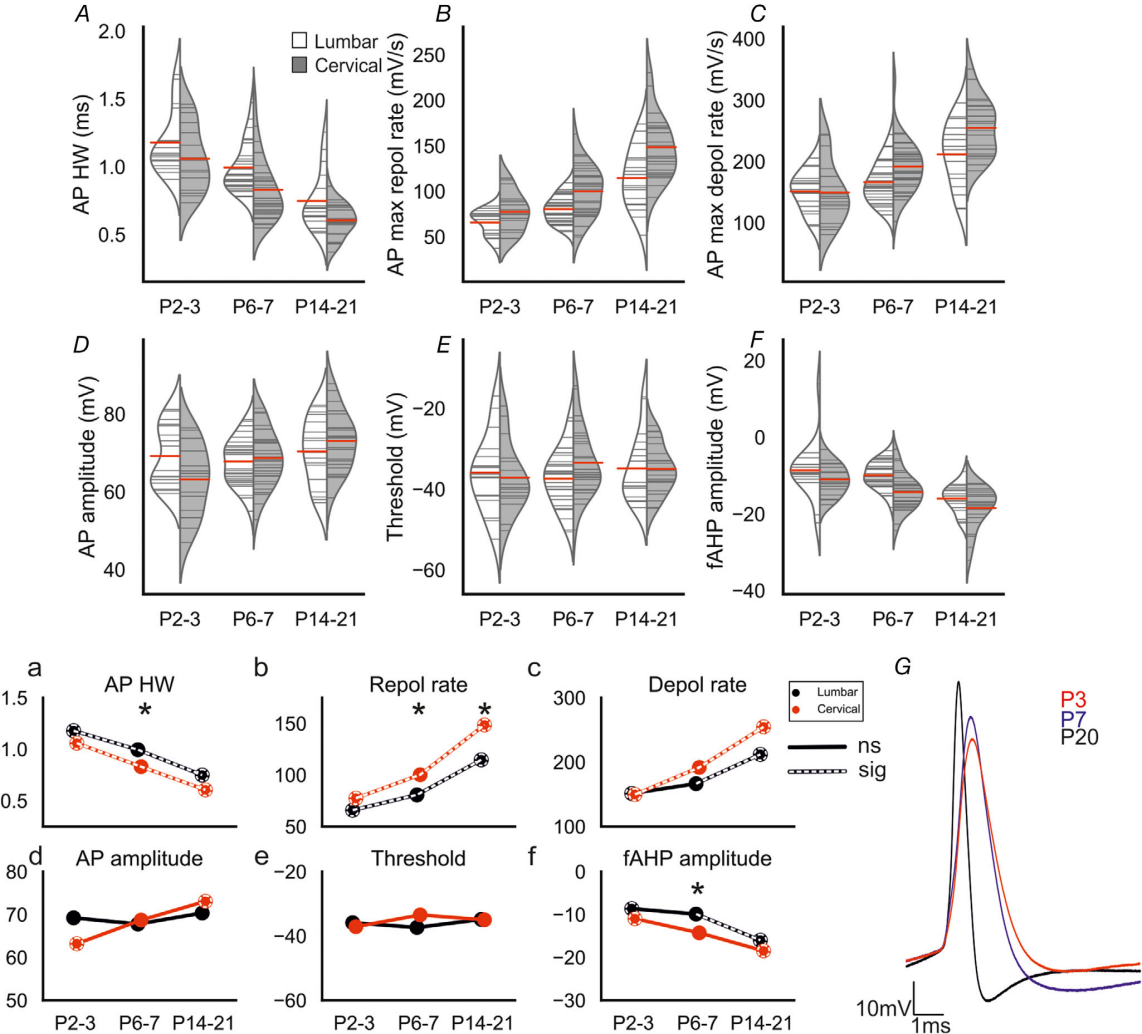
Postnatal development of action potential characteristics *A–F*, split violin plots showing the changes in action potential characteristics with development for lumbar (white) and cervical (grey) motoneurons. (*a*–*f*) Joint‐plots of means illustrating the developmental profile of each measure. Overlaid white dashed lines represent statistically significant changes between age groups. Dashed circles represent statistically significant difference between P2–3 and P14–21. * represents a statistically significant difference (*p* < 0.05) between regions for a particular age group. (*G*) Overlaid traces from representative motoneurons (cervical) from each of the age groups. Each trace was averaged from 15–30 action potentials with the cells’ resting membrane potential held at ‐65 mV. Mann–Whitney U tests with Holm–Sidak corrected *P* values used for pairwise comparisons. Cell numbers: lumbar & cervical P2–3, *n* = 17; lumbar & cervical P6–7, *n* = 30; lumbar P14–21, *n* = 18, cervical P14–21, *n* = 22. [Color figure can be viewed at wileyonlinelibrary.com]

A key indicator of functional maturation is AP (and afterpotential) morphology. The fast spike can be characterized by the half width, rate of rise and fall, threshold, amplitude, and depth of fall or fAHP. To assess changes in these characteristics we evoked single APs using 20 ms current pulses while the RMP was held at ‐65 mV. There was a significant decrease in AP HW for both lumbar (Spearman's ρ = ‐0.620, *p* = 5e‐07) and cervical motoneurons (Spearman's ρ = ‐0.792, *p* = 2e‐12, Fig. 2 *A, a*). In lumbar motoneurons the reduction in half width occurred at each time point (P2–3 = 1.2 ± 0.2 ms *vs*. P6–7 = 1.0 ± 0.2 ms, *p* = 0.015; P6–7 *vs*. P14–21 = 0.7 ± 0.2 ms, *p* = 0.001). The decrease between P2–3 and P6–7 was associated with an increase in the rate of repolarization (P2–3 = 66 ± 14 mV/s *vs*. P6–7 = 81 ± 15 mV/s, *p* = 0.032, Fig. 2 *B, b*) – there was no difference in the maximum depolarization rates between these age groups (P2–3 = 152 ± 33, P6–7 = 166 ± 34, *p* = 0.44, Fig. 2 *C, c*). Between P6–7 and P14–21, however, there was an increase in both the maximum rates of depolarization (P14–21 = 212 ± 53 mV/s, *p* = 0.032) and repolarization (P14–21 = 114 ± 34 mV/s, *p* = 0.002). These data suggest that delayed rectifier potassium channels mature earlier than voltage‐gated sodium channels.

AP HW also decreased in cervical motoneurons between P2–3 (1.1 ± 0.2 ms) and P6–7 (0.8 ± 0.2 ms, *p* = 0.009) and between P6–7 and P14–21 (0.6 ± 0.1, *p* = 0.001). Unlike lumbar motoneurons, both maximum rate of depolarization (P2–3 = 150 ± 51 mV/s *vs*. P6–7 = 192 ± 44 mV/s, *p* = 0.032; P6–7 *vs*. P14–21 255 ± 46 mV/s, *p* = 2e‐04) and repolarization (P2–3 = 77 ± 22 mV/s *vs*. P6–7 = 100 ± 28 mV/s, *p* = 0.035; P6–7 *vs*. P14–21 149 ± 34.7 mV/s, *p* = 7e‐05) increased throughout this period and thus contributed to the reduction in AP HW between each age group. These data suggest that both delayed rectifier potassium and voltage‐gated sodium channels mature throughout this period in cervical motoneurons.

At P2–3 no differences between cervical and lumbar motoneurons were observed in AP HW (*p* = 0.34), or maximum rate of depolarization (*p* = 0.32) or repolarization (*p* = 0.55). At P6–7, cervical motoneurons had shorter duration APs than lumbar motoneurons mainly due to a higher rate of repolarization (AP HW: *p* = 0.007, maximum rate of depolarization: *p* = 0.071 and maximum rate of repolarization: *p* = 0.023). There was no difference between regions in the P14–21 group for AP HW (*p* = 0.151), despite a higher maximum rate of repolarization in cervical motoneurons: *p* = 0.035). Overall, these results suggest significant changes in the AP HW, with cervical motoneurons developing earlier than lumbar motoneurons.

We next assessed changes in AP amplitude by measuring the difference between peak voltage and voltage threshold. AP amplitude increased only in cervical lumbar motoneurons (lumbar: Spearman's ρ = ‐0.0001, *p* = 0.99; cervical: Spearman's ρ = 0.375, *p* = 0.005, Fig. 2 *D, d*).

There was a gradual increase in AP amplitude in the cervical cord as there was no difference between P2–3 (63 ± 9 mV) and P6–7 (69 ± 7.4 mV, *p* = 0.55) or P6–7 and P14–21 (73 ± 7.7 mV, *p* = 0.52); however, P14–21 was significantly greater than P2–3 (*p* = 0.037). These data suggest that AP conductances mature throughout this period in cervical motoneurons.

Because spike amplitude is measured as the difference between threshold and peak, threshold changes could contribute to changes in amplitude. However, we found no correlation between age and AP threshold in either region (lumbar: Spearman's ρ = 0.062, *p* = 0.65; cervical: Spearman's ρ = 0.148, *p* = 0.29; Fig. 2 *E, e*, *G*). The apparent lack of developmental change for AP threshold suggests that development of Na^+^ channels may not contribute as much as later activated channels to the development of spike morphology.

#### Fast afterhyperpolarization

The amplitude of the fAHP (measured from threshold to trough) increased with age in both regions (lumbar: Spearman's ρ = ‐0.511, *p* = 7e‐05; cervical: Spearman's ρ = ‐0.611, *p* = 1e‐06; Fig. 2 *F, f*). In lumbar motoneurons, there was no difference in amplitude between P2–3 (‐8.6 ± 7.5 mV) and P6–7 (‐9.9 ± 3.5 mV, *p* = 0.66), but it increased between P6–7 and P14–21(‐16 ± 3.6 mV, *p* = 7e‐05). In cervical motoneurons, there was no difference in amplitude between P2–3 (‐11 ± 4.1 mV) and P6–7 (‐14 ± 4.8 mV, *p* = 0.105), or P6–7 and P14–21 (19 ± 5.5 mV, *p* = 0.104); however, there was an increase between P2–3 and P14–21 (*p* = 7e‐04). The only difference between spinal regions was a significantly greater fAHP in cervical motoneurons compared with lumbar motoneurons at P6–7 (P6–7, *p* = 0.002; P2–3, *p* = 0.67; P14–21, *p* = 0.66). This is further evidence that motoneuron voltage‐gated potassium channels involved in repolarization develop significantly postnatally in both regions, and that the process occurs earlier in cervical motoneurons.

### Postnatal development of motoneuron transition properties: afterpotentials

**Figure 3:**
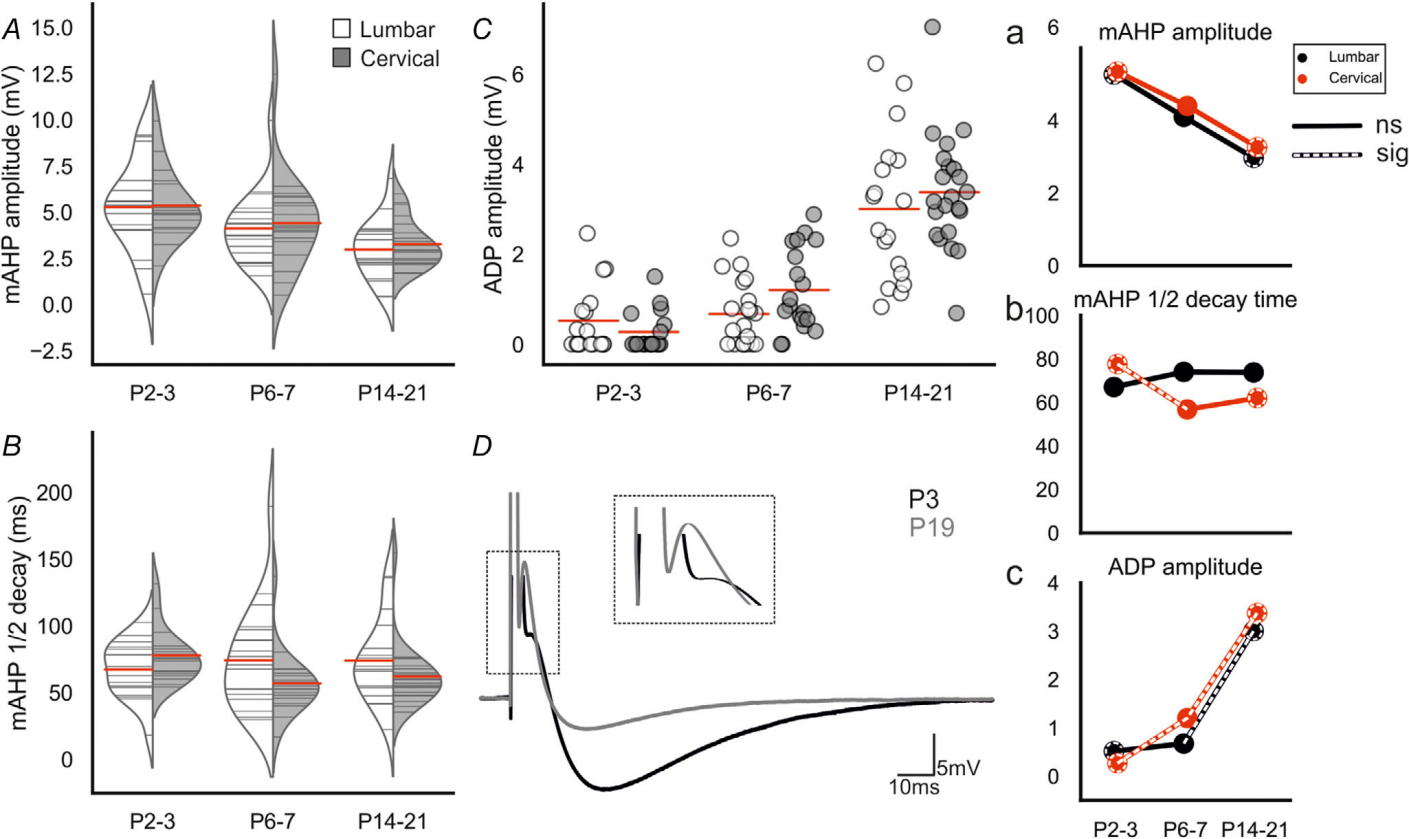
Postnatal development of afterpotentials *A–B*, split violin plots showing the changes in mAHP amplitude and mAHP half decay time at each age for lumbar (white) and cervical (grey) motoneurons. The mAHP amplitude was measured from baseline (all cells held at ‐65 mV) to the most negative point of the mAHP. Half decay time was measured from the mAHP peak to baseline. *C*, strip‐plots showing development of ADP amplitude with age. *D*, averaged trace (15–30 sweeps) of the action potential evoked with a 1 ms square current pulse for a representative motoneuron (cervical) at P3 (black) and P19 (grey). Action potential rising phases are truncated. ADP amplitude was measured from the most negative point of the fAHP to the peak of the ADP; see inset for expanded ADPs. (*a*–*c*) Joint‐plots illustrating the developmental profile of each measure. Overlaid white dashed lines represent statistically significant changes between age groups. Dashed circles represent statistically significant difference between P2–3 and P14–21. * represents a statistically significant difference (*p* < 0.05) between regions for a particular age group. Mann–Whitney U tests with Holm–Sidak corrected *P* values used for pairwise comparisons. Cell numbers: lumbar & cervical P2–3, *n* = 17; lumbar: *n* = 20, cervical: *n* = 19; lumbar P14–21, *n* = 18, cervical P14–21, *n* = 22. [Color figure can be viewed at wileyonlinelibrary.com]

AP afterpotential characteristics contribute to the repetitive firing properties of motoneurons (Granit *et al*. 1963a; Kernell, 1979). Slower afterpotentials, such as the mAHP and ADP, contribute significantly to repetitive firing capabilities of motoneurons, and reflect motoneuron type (Granit *et al*. 1963b; Kernell, 1972; Kernell & Monster, 1982). The mAHP amplitude (measured from RMP to trough, Fig. 3 *A, a*) significantly reduced with development in motoneurons of both regions (lumbar: Spearman's ρ = ‐0.382, *p* = 0.004; cervical: Spearman's ρ = ‐0.417, *p* = 0.0018). In lumbar motoneurons, the decrease was slow, as there was no difference between P2–3 (5.3 ± 2.4 mV) and P6–7 (4.1 ± 1.9 mV, *p* = 0.041) or P6–7 and P14–21 (3.0 ± 1.6 mV, *p* = 0.52), but P14–21 was smaller than P2–3 (*p* = 0.038). The same pattern of development was observed in cervical motoneurons (P2–3 = 5.4 ± 1.9 mV *vs*. P6–7 = 4.4 ± 2.6 mV, *p* = 0.53; P6–7 *vs*. P14–21 = 3.3 ± 1.3 mV, *p* = 0.28; P2–3 *vs*. P14–21, *p* = 0.002). Moreover, there were no differences between cervical and lumbar motoneuron mAHPs at any of the age groups (P2–3, *p* = 0.99; P6–7, *p* = 0.89; P14–21, *p* = 0.99).

The mAHP half decay time was reduced with age, but only in cervical motoneurons (lumbar: Spearman's ρ = ‐0.005, *p* = 0.970; cervical: Spearman's ρ = ‐0.398, *p* = 0.003, Fig. 3 *B, b*). This was due to a decrease between P2–3 (77 ± 20 ms) and P6–7 (56 ± 24 ms, *p* = 0.006) as there was no difference between P6–7 and P14–21 (62 ± 23 ms, *p* = 0.92).

#### Afterdepolarization

The ADP amplitude (measured from the trough of the fAHP to the peak of the subsequent depolarization, if present) increased during development in both regions (lumbar: Spearman's ρ = 0.623, *p* = 5e‐07; cervical: Spearman's ρ = 0.832, *p* = 1e‐14; Fig. 3 *C, c, D*). In fact, 41% of all motoneurons (regardless of region) expressed an ADP at P2–3 (14/34), with this proportion increasing to 77% at P6–7 (30/39), and 100% of motoneurons at P14–21 (43/43). In the lumbar spinal cord, 47% (8/17) of motoneurons expressed an ADP at P2–3, 65% (13/20) at P6–7, and 100% (18) at P14–21. However, there was no difference between mean ADP amplitude between P2–3 (0.5 ± 0.8 mV) and P6–7 (0.7 ± 0.7 mV, *p* = 0.58). Between P6–7 and P14–21, the ADP amplitude in lumbar motoneurons increased to 3.0 ± 1.6 mV (*p* = 1e‐04). In the cervical spinal cord at P2–3, the proportion of motoneurons expressing an ADP was 41% (7/17) and the amplitude 0.3 ± 0.4 mV. The proportion increased to 89% (17/19) and amplitude 1.2 ± 0.9 mV at P6–7 (*p* = 0.004) and to 100% (22) and 3.3 ± 1.3 mV between P6–7 and P14–21 (*p* = 8e‐05). There were no differences in ADP amplitude between lumbar and cervical motoneurons at P2–3 (*p* = 0.58), P6–7 (*p* = 0.16), or P14–21 (*p* = 0.58).

Given the importance of ADPs in generating high initial firing frequencies and doublets, these data suggest that development of ADPs enables motoneurons to fire at higher initial frequencies as they mature. Furthermore, this increase occurred earlier in cervical motoneurons, suggesting that development of high initial firing frequencies would develop earlier than in lumbar motoneurons (see below).

### Postnatal development of motoneuron transition properties: sag potentials

**Figure 4:**
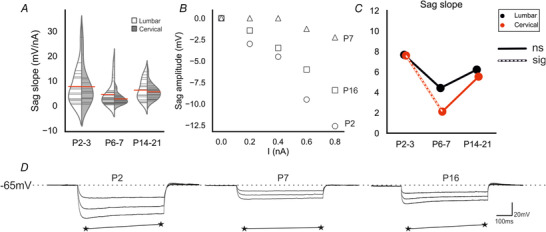
Postnatal development of sag potential *A*, split violin plots showing the changes in sag slope with development. *B*, plots of sag voltage *vs*. hyperpolarizing current injected for three motoneurons illustrate the developmental changes in sag conductance (inverses of slope). *C*, joint‐plots of sag slope means illustrating the developmental profile. Overlaid white dashed lines represent statistically significant changes between age groups. *D*, examples of voltage responses to ‐0.4, ‐0.7 and ‐1 nA current injections in representative motoneurons from each age group. The sag amplitude was measured as the difference between the negative voltage peak at the start of the 500 ms pulse and the steady state at the end (stars). Tukey's *post hoc* test was used for pairwise comparisons following two‐way ANOVA. Cell numbers: lumbar & cervical P2–3, *n* = 17; lumbar & cervical P6–7, *n* = 30; lumbar P14–21, *n* = 18, cervical P14–21, *n* = 22. [Color figure can be viewed at wileyonlinelibrary.com]

Sag potentials mediated by Ih can also be considered to be transitional properties, as they contribute to motoneuron firing and are modulated by various neurotransmitters associated with input amplification (Ito & Oshima, 1965; Berger *et al*. 1996). We assessed the slope of the sag potential, which is a reliable representative of the Ih conductance change (Fig. 4 *A–D*). There was a significant effect of age in the two‐way ANOVA (*F* = 7.9, *p* = 5e‐04). However there was no effect of region (*F* = 1.6, *p* = 0.20) and no interaction between the independent variables (IVs, *F* = 0.3, *p* = 0.6). In cervical motoneurons there was a reduction between P2–3 (‐6.7 ± 8.0 mV/nA) and P6–7 (2.6 ± 2.1 mV/nA, *p* = 0.022), but no change between P6–7 and P14–21 (5.5 ± 2.9 mV/nA, *p* = 0.23). There was no difference between any of the ages in lumbar motoneurons (P2–3 = 7.7 ± 7.3 mV/nA *vs*. P6–7 = 4.4 ± 4.8 mV/nA, *p* = 0.64; P6–7 *vs*. P14–21 = 6.1 ± 4 mv/nA, *p* = 0.9; P2–3 *vs*. P14–21, *p* = 0.9), nor was there a difference between regions at any age (P2–3, *p* = 0.9, P6–7, *p* = 0.34, P14–21, *p* = 0.9).

### Postnatal development of motoneuron firing properties: minimum current for repetitive firing (I_min_)

For function, motoneurons must fire repetitive trains of APs. The frequencies of firing are graded, with increased synaptic (or injected) current leading to higher rates of firing. By plotting the frequency of firing *vs*. the current injected, various key measures can be quantified, including the minimum current needed for repetitive firing (I_min_), the maximum firing rate obtainable (F_max_) and the slopes for initial, steady state, and overall relationships.

Consistent with the observed increase in motoneuron WCC (i.e. size) and decrease in membrane resistance, there was a significant increase in I_min_ over postnatal development in motoneurons of both regions (lumbar: Spearman's ρ = 0.548, *p* = 1e‐05; cervical: Spearman's ρ = 0.708, *p* = 3e‐09). In lumbar motoneurons, there was no change in I_min_ between P2–3 (0.45 ± 0.38 nA) and P6–7 (0.52 ± 0.27 nA, *p* = 0.35), but it increased between P6–7 and P14–21 (1.50 ± 1.11 nA, *p* = 0.002). In cervical motoneurons, I_min_ increased between P2–3 (0.37 ± 0.20 nA) and P6–7 (0.81 ± 0.45 nA, *p* = 0.004), but not between P6–7 and P14–21 (1.29 ± 0.67 nA, *p* = 0.052). There was no difference observed between regions at P2–3 (*p* = 0.99), P6–7 (*p* = 0.054), or P14–21(*p* = 0.99).

### Postnatal development of motoneuron firing properties: maximal spike output

**Figure 5:**
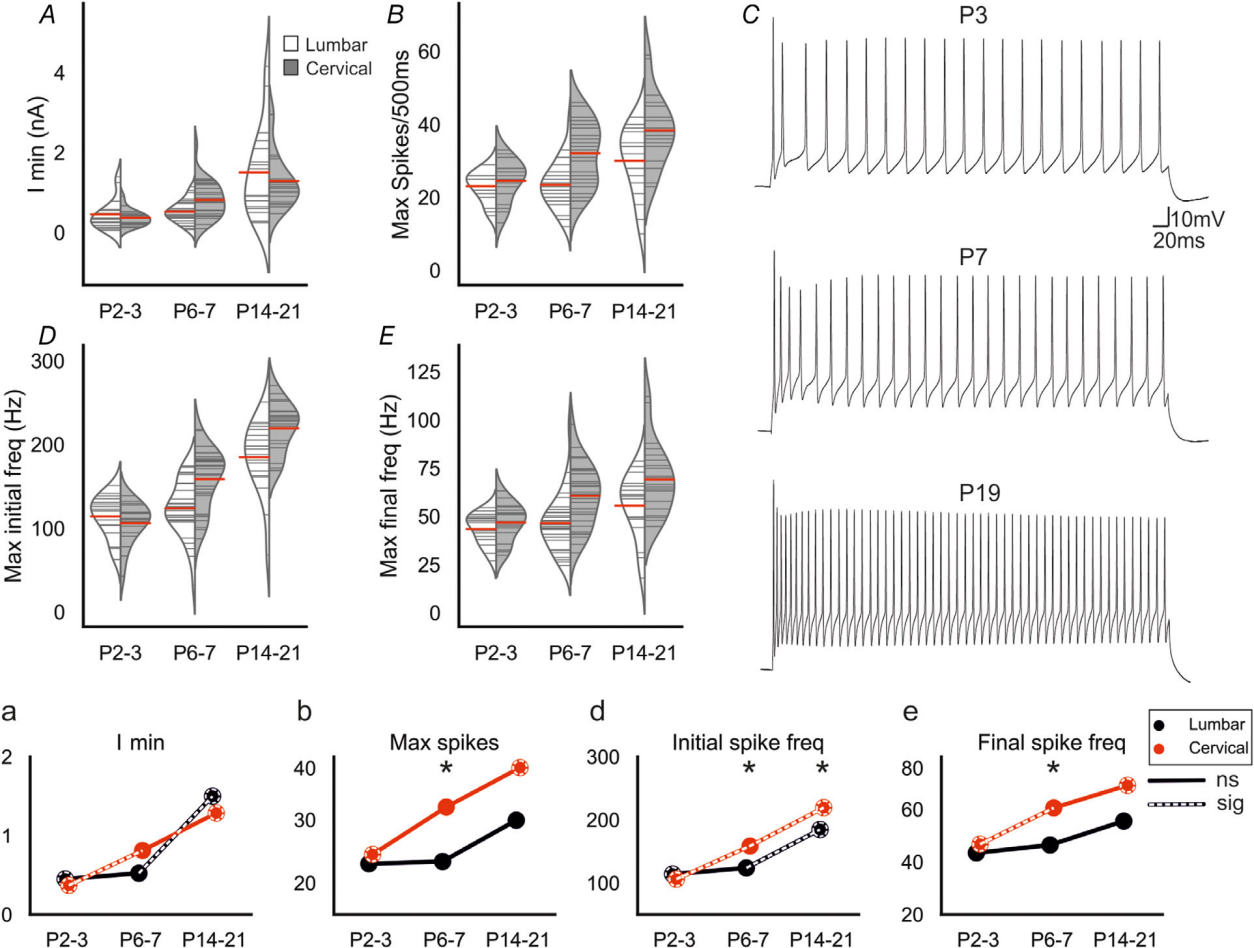
Postnatal development of repetitive firing *A–B*, violin plots showing the minimum current required for repetitive firing and maximum number of spikes in lumbar (white) and cervical (grey) motoneurons at each age group. *C*, representative traces of maximum firing of neurons from each age group, illustrating the increased ability of motoneurons to produce high frequency trains of action potentials as they mature. Depolarizing current pulses (500 ms) of increasing magnitude were injected until the cell reached its maximum firing frequency or depolarizing block. *D*, max initial firing frequency was calculated from the first two action potentials at maximum firing rate. *E*, the final firing frequency was calculated from the last two action potentials at maximum firing rates. (*a*–*e*) Joint‐plots of means illustrating the developmental profile for each measure. Overlaid white dashed lines represent statistically significant changes between age groups. Dashed circles represent statistically significant difference between P2–3 and P14–21. * represents a statistically significant difference (*p* = <0.05) between regions for a particular age group. Mann–Whitney U (*a*, *b*, *e*) or *t* tests (*d*) with Holm–Sidak corrected *P* values used for pairwise comparisons. Cell numbers: lumbar & cervical P2–3, *n* = 17; lumbar & cervical P6–7, *n* = 30; lumbar P14–21, *n* = 18, cervical P14–21, *n* = 22. [Color figure can be viewed at wileyonlinelibrary.com]

In order to assess motoneuron firing capabilities throughout development, 500 ms current pulses of increasing magnitude were injected into cervical and lumbar motoneurons until the maximum firing frequency (F_max_) was attained. Interestingly, for maximum spike output there was a significant increase in cervical but not lumbar motoneurons (lumbar: Spearman's ρ = 0.248, *p* = 0.070; cervical: Spearman's ρ = 0.567, *p* = 9e‐06, Fig. 5 *B, b, C*). For other measures, there were significant increases in both lumbar and cervical motoneurons, including maximum initial frequency (lumbar: Spearman's ρ = 0.526, *p* = 4e‐05; cervical: Spearman's ρ = 0.841, *p* = 3e‐14, Fig. 5 *D, d*) and maximum final frequency (lumbar: Spearman's ρ = 0.282, *p* = 0.038; cervical: Spearman's ρ = 0.528, *p* = 4e‐05, Fig. 5 *E, e*).

In lumbar motoneurons, maximum initial frequency increased between P6–7 and P14–21 (maximum initial freq: P6–7 = 124 ± 34 Hz, P14–21 = 185 ± 44 Hz, *p* = 2e‐05). However, there was no change between these ages for number of spikes per 500 ms current pulse (P6–7 = 23 ± 6.5, P14–21 = 30 ± 9.4, *p* = 0.076) or maximum final frequency (P6–7 = 47 ± 15 Hz, P14–21 = 56 ± 17 Hz, *p* = 0.15). There was no change between P2–3 and P6–7 for any of the measures (number of spikes per 500 ms current pulse P2–3 = 23 ± 4.2 Hz, *p* = 0.9; maximum initial frequency: P2–3 = 114 ± 25 Hz, *p* = 0.5; maximum final frequency: P2–3 = 44 ± 8.1 Hz, *p* = 0.8).

In cervical motoneurons, maximum initial frequency increased between P2–3 (106 ± 25 Hz) and P6–7 (159 ± 35 Hz, *p* = 2.2e‐05) and between P6–7 and P14–21 (219 ± 30 Hz, *p* = 3e‐07). There was also an increase between P2–3 and P6–7 for final frequency (P2–3 = 47 ± 9.9, P6–7 = 61 ± 16, *p* = 0.043), but not maximum spike number (P2–3 = 25 ± 5.8, P6–7 = 32 ± 8.7, *p* = 0.082). There was no difference between P6–7 and P14–21 for maximum final frequency (P14–21 = 69 ± 19 Hz, *p* = 0.51) or maximum spike number (P14–21 = 38 ± 9.8, *p* = 0.17).

Comparisons between cervical and lumbar motoneurons at P2–3 showed no differences for any of the maximum firing measurements (number of spikes per 500 ms current pulse, *p* = 0.73, maximum initial frequency, *p* = 0.52; maximum final frequency, *p* = 0.71). At P14–21, cervical motoneurons had greater maximum initial frequencies (*p* = 0.025), but maximum spike output (*p* = 0.11) and final firing frequency (*p* = 0.24) were not different between regions. However, at P6–7 cervical motoneuron values were greater than lumbar motoneurons for all measures (number of spikes per 500 ms current pulse: *p* = 0.003; maximum initial frequency: *p* = 0.001; maximum final frequency: *p* = 0.008). Thus, repetitive firing parameters seem to mature earlier in cervical than in lumbar motoneurons.

Overall, motoneuron repetitive firing function matured over postnatal development, with cervical motoneurons increasing their firing capacity earlier than lumbar motoneurons. These results are consistent with the developmental profile of AP characteristics described above.

### Postnatal development of motoneuron firing properties: excitability

**Figure 6:**
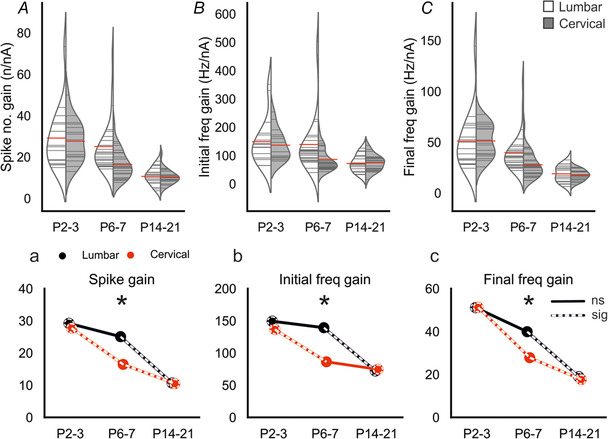
Postnatal development of cervical and lumbar motoneuron excitability *A–C*, split violin plots showing postnatal changes in the slope of the linear portion of an f‐I plot. (*a*–*c*) Joint‐plots of means illustrating developmental profile of each measure. Overlaid white dashed lines represent statistically significant changes between age groups. Dashed circles represent statistically significant difference between P2–3 and P14–21. * represents a statistically significant difference (*p* = <0.05) between regions for a particular age group. Mann–Whitney U with Holm–Sidak corrected *P* values used for pairwise comparisons. Cell numbers: lumbar & cervical P2–3, *n* = 17; lumbar & cervical P6–7, *n* = 30; lumbar P14–21, *n* = 18, cervical P14–21, *n* = 22. [Color figure can be viewed at wileyonlinelibrary.com]

Next, we assessed the changes in motoneuron excitability with development. This was done by measuring the slope of the linear portion of the relationships between the current injected and each of: number of spikes per 500 ms pulse (spike slope); instantaneous frequency of the first two spikes (initial firing frequency); or instantaneous frequency of the last two spikes (final firing frequency or steady state, Fig. 6 *A–C, a–c*). For all these measures, there were decreases with development (spike number f‐I slope‐ lumbar: Spearman's ρ = ‐0.699, *p* = 2e‐09; cervical: Spearman's ρ = ‐0.706, *p* = 3e‐09; initial frequency gain‐ lumbar: Spearman's ρ = ‐0.550, *p* = 1e‐05; cervical: Spearman's ρ = ‐0.493, *p* = 0.0001; final frequency gain‐lumbar: Spearman's ρ = ‐0.681, *p* = 1e‐08; cervical: Spearman's ρ = ‐0.712, *p* = 2e‐09).

For lumbar motoneurons, there was no change in the spike number f‐I slope between P2–3 (29 ± 15 spikes/nA) and P6–7 (25 ± 13 spikes/nA, *p* = 0.74). However, there was a significant decrease between P6–7 and P14–21 (11 ± 4.1 spikes/nA, *p* = 2e‐06). The spike slope in cervical motoneurons decreased between all age groups (P2–3 = 30 ± 10 spikes/nA *vs*. P6–7 = 17 ± 6.8 spikes/nA, *p* = 0.002; P6–7 *vs*. P14–21 = 11 ± 3.6 spikes/nA, *p* = 0.003). There was no difference in spike slope between lumbar and cervical motoneurons at P2–3 (*p* = 0.95) or P14–21 (*p* = 0.89); however, it was lower in cervical motoneurons at P6–7 (*p* = 0.002).

As with spike number slope, initial firing frequency slope decreased in lumbar motoneurons between P6–7 (139 ± 102 Hz/nA) and P14–21 (72 ± 30 Hz/nA, *p* = 0.003), but there was no difference between P2–3 (149 ± 84 Hz/nA) and P6–7 (*p* = 0.90). In cervical motoneurons, there was a decrease in the slope of the initial frequency gain between P2–3 (137 ± 53 Hz/nA) and P6–7 (86 ± 37 Hz/nA, *p* = 0.003), but not between P6–7 and P14–21 (75 ± 29 Hz/nA, *p* = 0.90). Furthermore, as with the spike slope, initial frequency gain was lower in cervical than lumbar motoneurons at P6–7 (*p* = 0.015), but there was no difference at P2–3 (*p* = 0.97) or P14–21(*p* = 0.90).

For the slope of the instantaneous frequency of the final two spikes, in lumbar motoneurons there was a decrease between P6–7 (40 ± 19 Hz/nA) and P14–21 (18 ± 7.9 Hz/nA, *p* = 1e‐05), but not between P2–3 (51 ± 29 Hz/nA) and P6–7 (*p* = 0.31). In cervical motoneurons, the steady state slope decreased significantly between all age groups (P2–3 = 51 ± 16 Hz/nA *vs*. P6–7 = 28 ± 12 Hz/nA, *p* = 0.0001; P6–7 *vs*. P14–21 = 18 ± 5.1 Hz/nA, *p* = 0.0004). Cervical motoneurons had a lower slope for steady state gain at P6–7 compared with lumbar motoneurons (*p* = 0.012), but there was no difference between regions at P2–3 (*p* = 0.74) or P14–21 (*p* = 0.74).

In summary, for almost all measures of repetitive firing, it can be seen that at P2–3 lumbar and cervical motoneurons are the same, but there is a reduction in excitability for cervical motoneurons at P6–7 (Fig. 6 *a–c*). In lumbar motoneurons, this reduction is seen later, between P6–7 and P14–21.

### Principal component analysis

**Figure 7:**
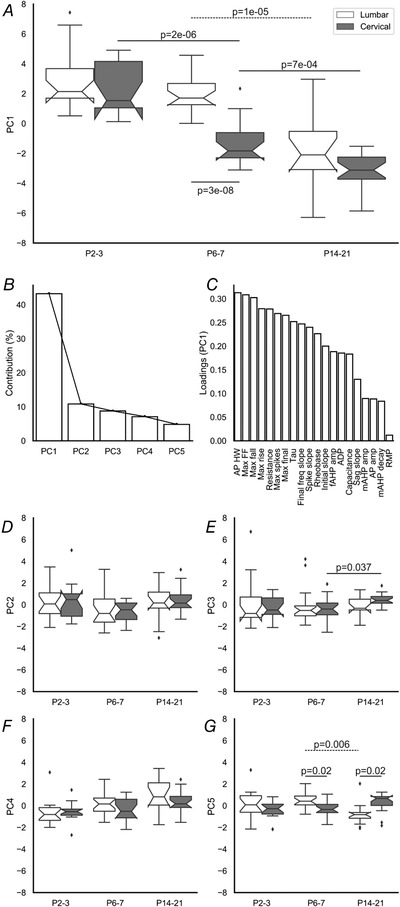
Principal component analysis of the development of motoneuron electrophysiological properties *A*, Box‐and‐whisker plots show the quartiles (box) and distribution (whiskers) of all data points. Outliers are shown by diamond shapes. Continuous lines show differences between ages in cervical region and compartments within an age group. Dashed lines show differences between ages in lumbar motoneurons. *B*, Scree plot showing that *C*, the first PC accounted for 43.2% of the variation. Loading features for PC1 were maximal firing and excitability parameters. *D–G*, box plots showing PCs 2–5 at each age for lumbar (white) and cervical (grey) motoneurons. Mann–Whitney U (*D*–*G*) or *t* tests (*A*) with Holm–Sidak corrected *P* values were used for pairwise comparisons. Cell numbers: lumbar & cervical P2–3, *n* = 17; lumbar & cervical P6–7, *n* = 30; lumbar P14–21, *n* = 18, cervical P14–21, *n* = 22.

Analysis of the individual variables above suggests that there is a significant effect of age on the development of motoneuron membrane properties and firing characteristics. Additionally, for many firing properties, we found a difference in the developmental profile between lumbar and cervical motoneurons, with cervical motoneurons maturing earlier than lumbar motoneurons. Due to the high dimensionality of the data, we used principal component analysis to assess overall changes in motoneuron properties between ages and regions. Analysis of PC1 (Fig. 7 *A*) revealed that there was a negative shift in PC1 for both lumbar (Spearman's ρ = ‐0.608, *p* = 2e‐06) and cervical motoneurons (Spearman's ρ = ‐0.797, *p* = 1e‐11). PC1 accounted for 43.2% of the variance (Fig. 7 *B*). The parameters with the greatest loadings for PC1 were maximum firing, fast AP and excitability characteristics (Fig. 7 *C*), which, when individually assessed (see above), had the greatest changes with development and greatest differences between cervical and lumbar compartments. In lumbar motoneurons, PC1 did not change between P2–3 (2.93 ± 2.03) and P6–7 (2.11 ± 1.21, *p* = 0.44); however, there was a significant decrease subsequently (P14–21 = ‐1.76 ± 1.77, *p* = 1e‐05). In contrast, in cervical motoneurons, PC1 decreased between each age group (P2–3 = 2.60 ± 1.77 *vs*. P6–7 = ‐1.32 ± 1.47, *p* = 2e‐06; P6–7 *vs*. P14–21 = ‐3.15 ± 1.13, *p* = 7e‐04), again suggesting that firing properties develop earlier than in lumbar motoneurons. Of note, there was no difference in PC1 between regions at P2–3 (*p* = 0.82) or P14–21 (*p* = 0.16), but cervical motoneuron values were significantly more negative at P6–7 (*p* = 3e‐08), in keeping with their earlier maturation.

Further principal components (PCs 2–5, Fig. 7 *D–G*) individually accounted for comparatively little variation, and had greater loadings from passive membrane properties (see analysis notebook link in *Methods* for loadings). In summary, PC2 (10.8%) did not change with development (Spearman's ρ: lumbar = ‐0.010, *p* = 0.947; cervical = 0.111, *p* = 0.430), and although PCs 3–5 showed weak correlations with age, none accounted for more than 8.7% of the variance (PC3 (8.7%) – Spearman's ρ lumbar = 0.146, *p* = 0.311 and cervical = 0.307, *p* = 0.025; PC4 (7.1%) – Spearman's ρ lumbar = 0.459, *p* = 8e‐04 and cervical = 0.321, *p* = 0.018; PC5 (4.8%) – Spearman's ρ lumbar = 0.329, *p* = 0.019 and cervical = 0.375, *p* = 0.005). Of note, there was a small increase between P6–7 (‐0.33 ± 1.03) and P14–21(0.45 ± 0.53, *p* = 0.037) for PC3 in cervical motoneurons and lumbar motoneurons for PC5 (P6–7 = 0.08 ± 2.29, *p* = 0.006). Additionally, there were differences between lumbar and cervical regions at P6–7 (cervical = ‐0.32 ± 0.71, lumbar = 0.47 ± 0.74, *p* = 0.024) and P14–21 (cervical = 0.34 ± 0.81, lumbar = ‐0.75 ± 1.04, *p* = 0.025) for PC 5.

In summary, these data further support suggestions from analyses of individual firing characteristics that lumbar and cervical motoneurons are similar at birth and undergo significant postnatal development. Importantly, they also provide further evidence for earlier postnatal development of cervical compared with lumbar motoneurons.

## Discussion

Altricial mammals are born in an immature state, and their nervous systems and musculoskeletal systems must develop such that there is sufficient motor independence prior to the time of weaning. These two systems, which develop hand in hand, are connected by motoneurons and proprioceptive afferents. Here, we ask how the properties of motoneurons develop during this period, and ask whether the evidence supports cell autonomous or circuit factors as being the leading instigators for this development. To do this, we compare lumbar and cervical spinal motoneuron properties through this postnatal period, examining passive, transition and repetitive firing properties from a stage where the animal is virtually immobile (P2–3) to the point when motor function matures, just prior to weaning (P14–21). We show that there is ongoing development of both cervical and lumbar motoneuron properties throughout this period and that despite the fact that cervical motoneurons are embryonically born prior to lumbar motoneurons (Nornes & Das, 1974), their properties at birth are similar. In the first 3 weeks of postnatal life, the properties of both sets mature, but those of cervical motoneurons develop earlier than those of lumbar motoneurons. This development correlates with the arrival of descending axons, which is known to be crucial to the maturation of spinal sensorimotor circuits. We therefore suggest that maturation of these circuits contributes to development of motoneuron firing properties.

### The critical period: postnatal development of motoneuron properties continues into the third postnatal week

Weight bearing and fundamental aspects of motor control are established to a degree by P10–12, but it is clear that motor functional output in rodents does not mature until the third postnatal week (Altman & Sudarshan, 1975). Indeed, anatomical studies show that developmental organization of membrane proteins and synaptic input to spinal motoneurons begin to reach maturity in the third week postnatally in rodents (Wilson *et al*. 2004; Jean‐Xavier *et al*. 2017; Smith *et al*. 2017). A profile of changes in passive properties and firing characteristics of motoneurons has been described for rodent hypoglossal and genioglossal motoneurons from birth to adulthood, but equivalent studies in the spinal cord have been limited to ages younger than P12 (Fulton & Walton, 1986; Nunez‐Abades *et al*. 1993; Berger *et al*. 1996; Nakanishi & Whelan, 2010; Quinlan *et al*. 2011). Due to the ready decline in motoneuronal viability in slices after P10, early neonatal spinal cord preparations have become the dominant tool to study motoneuron physiology, and therefore much of our knowledge is limited to the first postnatal week in rodents and to the adult in cats (Kernell, 1999). Our recordings from spinal motoneurons in the cervical and lumbar spinal cord up to P21 confirm that maturation of electrophysiological properties continues into the third postnatal week, and demonstrates the importance of using older preparations to study neuronal properties.

Changes in motoneuron properties are dependent upon the expression and function of many membrane proteins. Although we did not study specific motoneuron ion channels, our results indicate that many channels undergo developmental changes in this critical period. During this time, WCC increases and input resistance is reduced, with a corresponding increase in I_min_. These changes are expected with growth of the motoneuron soma and dendritic tree, but also indicate that there is maturation of conductances that are active near resting potential (Fleshman *et al*. 1981; Vinay *et al*. 2000)_._ Analysis of AP morphology shows reduced half width due to increased rates of both depolarization and repolarization phases, suggesting that expression of Na^+^ (Barrett & Crill, 1980), Ca^2+^ (Hounsgaard & Mintz, 1988; Mynlieff & Beam, 1992; Viana *et al*. 1993a), and K^+^ channels undergoes maturation (Viana *et al*. 1993b). Thus, widespread changes to the motoneuron membrane occur during this period.

### Maturation of repetitive firing

A motoneuron's raison d’être is to ensure that muscle fibres contract appropriately to produce the behaviour, and to do so the motoneuron must be able to fire graded trains of APs. The maturation of spike properties, including increasing amplitude and reduced half width resulting from increasing rates of depolarization and repolarization, can facilitate faster firing (McCormick *et al*. 1985). There is also an increase in the expression and amplitude of the ADP, a potential likely dependent upon high voltage activated Ca^2+^ channels (Granit *et al*. 1963b; Kobayashi *et al*. 1997; Vinay *et al*. 2000). ADPs promote doublet firing in motoneurons, significantly increasing the rate and magnitude of muscle force generation (Parmiggiani & Stein, 1981; Sandercock & Heckman, 1997). The increase in ADP that we report here may underlie the increase of maximum initial firing frequency across this developmental period. These changes in APs and ADPs thus likely underpin the changes in motoneuron repetitive firing capabilities.

The mAHP is also an important factor in regulating the frequency of firing, or interspike interval (Granit *et al*. 1963a; Kernell & Monster, 1982; Bean, 2007; Deardorff *et al*. 2013). This afterpotential is mediated by small conductance Ca^2+^‐activated potassium channels (SK2 & SK3; Deardorff *et al*. 2013). We report a small developmental change in the mAHP amplitude in cervical, but not lumbar, motoneurons. Although greater changes in the mAHP might have been expected based upon the reported development in hypoglossal nuclei (Viana *et al*. 1994; Berger *et al*. 1996), our results are largely consistent with previous work in young spinal cord slices and brainstem slices throughout development (Carrascal *et al*. 2005; Nakanishi & Whelan, 2010; Quinlan *et al*. 2011).

The ability of motoneurons to fire trains of APs at high frequencies increased with development, while excitability, as measured by the gain of f‐I plots, decreased. This reduction in f‐I slope is likely related to the change in passive properties such as size of the soma and dendritic tree (Ulfhake & Cullheim, 1988; Ulfhake *et al*. 1988), and active properties such as potassium channels associated with spike repolarization (Gao & Ziskind‐Conhaim, 1998; Martin‐Caraballo & Greer, 2000). The combination of higher F_max_ and reduced f‐I slope leads to a broader range of input currents to which the motoneuron responds, an increase in signal‐to‐noise ratio of the response, and an enhancement in the tunability of motoneuron firing rates. It is perhaps intuitive that these properties continue developing throughout the third postnatal week and beyond, as the speed, force, and control of motor output continues to mature across this period (Altman & Sudarshan, 1975).

### Possibility of selection bias?

Could some of the differences in properties that we report result from selection bias? For example, SK2/3 expression and thus mAHP characteristics are different in different motoneuron types (i.e. those innervating fast *vs*. slow twitch muscle fibres, which correspond to large *vs*. small motoneurons (Deardorff *et al*. 2013). While in older preparations there is an inherent bias to record smaller motoneurons (because they survive; see Mitra & Brownstone (2012) where average input resistance was 123 mΩ), we were clearly not biased to smaller motoneurons: the mean input resistances we report in the oldest age group were approximately 30 mΩ. In fact, in this study, most motoneurons at P6–7 were under 50 mΩ and at P2–3 most were under 100 mΩ, both in the range of larger motoneurons. Furthermore, it seems that GFP expression decreases in Hb9::eGFP mice during this time period, persisting primarily in large motoneurons (and possibly even a specific subset of these). It is thus likely that we sampled the largest motoneurons in each age group. But there is also no doubt that motoneurons are growing during this period, and there is no way, at this point in time, to target neurons in younger mice that may be “destined” to grow up to be large motoneurons. That is, in order to reliably track the development of properties such as the mAHP, motoneuron types must be identified throughout development, which is a difficult proposition considering that motoneurons undergo significant diversification during postnatal development (Navarrete & Vrbová, 1993; Kanning *et al*. 2010).

### Possible factors leading to advanced maturation of cervical *vs*. lumbar motoneurons

Sequential rostro‐caudal development of many components of the nervous system has been observed both pre‐ and postnatally. During embryonic development, spinal motoneurons are born first in the cervical spinal cord, and then sequentially in the more caudal segments (Nornes & Das, 1974). This could mean that delayed postnatal maturation of motoneuron firing characteristics in the lumbar cord may be set from the point of neurogenesis. We find this unlikely to be the case, however, as there are few differences between diverse sets of motoneuron properties in cervical *vs*. lumbar regions at P2–3 (see, e.g. Fig. 7). However, by P6–7, cervical motoneurons had more mature properties than lumbar motoneurons. We therefore propose an alternative hypothesis suggesting that regional differences in states of motor circuit maturity and activity patterns during postnatal development underlie the differences seen between cervical and lumbar motoneuron firing characteristics.

The delayed arrival and maturation (including myelination and synaptic refinement) of supraspinal descending systems is perhaps the most obvious difference between the two regions during postnatal development (Donatelle, 1977; Gianino *et al*. 1999; Vinay *et al*. 2005). And following arrival of these systems, their termination patterns as well as their synaptic effects on spinal motoneurons also mature – with cervical effects thus preceding lumbar changes (Commissiong, 1983; Tanaka *et al*. 1992; Floeter & Lev‐Tov, 1993; Brocard *et al*. 1999b). These developmental changes are parallel to behavioural changes, as can be seen in the development of postural control (Skoglund, 1960; Brocard *et al*. 1999a), which occurs in a proximo‐distal gradient corresponding to the more caudal location of motor nuclei innervating distal muscles (Nicolopoulos‐Stournaras & Iles, 1983). Furthermore, normal development of sensory afferents and spinal premotor circuits are dependent upon descending innervation (Chakrabarty & Martin, 2011b; Smith *et al*. 2017), and these afferents themselves may affect motoneuron maturation (Woolley *et al*. 1999). The importance of these systems on motoneuron properties has been seen when descending tracts are prevented from growing: there is disruption of the development of inhibitory systems such as Renshaw cells and GABA‐pre circuits, and motoneuron hyperexcitability emerges (Martin, 2005; Chakrabarty *et al*. 2009; Chakrabarty & Martin, 2011a, b; Basaldella *et al*. 2015; Smith *et al*. 2017). We thus propose that while cell autonomous factors may play an important role, the differences in the states of maturation of cervical *vs*. lumbar motoneurons largely result from differences in the timing of local sensorimotor circuit development in the two regions, which is affected by innervation by descending systems.

## Conclusion

We show that between birth and weaning, cervical motoneuron properties mature earlier than those of lumbar motoneurons. We suggest that this maturation results from the development of circuits that engage these motoneurons. It has been shown that the properties of many different neuronal types mature in this postnatal period, including thalamocortical neurons (Warren & Jones, 1997), somatosensory cortical inhibitory neurons (Kinnischtzke *et al*. 2012), medial prefrontal cortex pyramidal neurons (Favuzzi *et al*. 2019), and auditory cortex pyramidal neurons (Oswald & Reyes, 2008), amongst others. While of course cell autonomous factors such as transcription factor expression respond to environmental cues such as morphogens to govern cell fate, including the fate to become a motoneuron (Jessell, 2000b; Dasen *et al*. 2008), the degree to which these molecules set a maturating course is not clear (Harb *et al*. 2016). In fact, many fundamental motoneuron properties can develop in motoneurons derived in a dish from embryonic stem cells (Miles *et al*. 2004), demonstrating the strength of intrinsic programmes in determining electrophysiological properties. From our study of geographically separated motoneurons, however, we argue that circuit factors play an important role in physiological maturation. That is, it could be argued that cell autonomous factors are sufficient for the acquisition of repetitive firing, and that motor circuit development and activity contribute to maturation. It seems likely that the combination of cell autonomous factors and circuit development are required to ultimately produce a mature, functional neuron.

## Additional information

### Competing interests

The authors have no competing interests to declare.

### Author contributions

C.C.S. conceptualized and designed the study, acquired and analysed the data; C.C.S. and R.M.B. interpreted the data and wrote the article.

Both the authors approve the final version of the article and agree to be accountable for all aspects of the work. The authors confirm that all persons designated as authors are qualified.

### Funding

This work was supported by Wellcome (110193), and RB is supported by Brain Research UK.

## Supporting information


Statistical Summary Document

## Data Availability

The following link provides access to a Github repository containing the electrophysiology data and a fully interactive analysis notebook. This notebook is an interactive version of the paper, with editable code ‘chunks’ available for each figure and statistical output. All programming language dependencies are embedded in the interactive paper so that figures/statistical analyses can be rerun in a web browser by clicking the link. *Interactive notebook link* https://mybinder.org/v2/gh/CalvinChad/Cervical_lumbar_development/master
