# Supplementary material for: Spinal motoneuron firing properties mature from rostral to caudal during postnatal development of the mouse
Source: J Physiol. 2020 Sep 16;598(23):5467–85. doi: 10.1113/JP280274 (PMC8436765; doi:10.1113/JP280274)
Supplement: Supplementary file 1 — Statistical Summary Document [file TJP-598-5467-s001.docx]

**Manuscript Title:** Spinal motoneuron firing properties mature from rostral to caudal during post-natal development of the mouse

**Authors:** Calvin C. Smith & Robert M. Brownstone

**Animal model used, if applicable:** Mouse, Hb9:: eGFP

**Underlying hypothesis:** Electrophysiological properties of cervical motoneurons develop prior to lumbar motoneurons

**Definitions of ‘n’:** ‘n’ is defined as one motoneuron

**Statistical summary table:**

| **Experimental question number*** | **Finding/ conclusion** | **Experimental location/ variable**  **e.g. cortex vs cerebellum or genotype** | **Mean** | **SD** | **n** | **P**** | **Units** | **Data comparisons**  **e.g. WT vs KO** | **Statistical test** | **Figure** |
| --- | --- | --- | --- | --- | --- | --- | --- | --- | --- | --- |
| 1. Is development of RMP different in cervical and lumbar motoneurons | Lumbar RMP correlated with age but No change between time points or segments | Age: P2-3,P6-7,P14-21  Segment: Lumbar, Cervical  L P2-3  L P6-7 L P14-21 C P2-3  C P6-7  C P14-21 | -65  -67  -65  -65  -67  -66 | 4.4  3.1  3.2  2.8  2.7  2.4 | 17  17  30  30  18  22 | Spearmans correlation coefficient: -0.074  Cervical RMP is not correlated with age (fail to reject H0) p=0.598  Spearmans correlation coefficient: -0.420  lumbar RMP is correlated with age(reject H0) p=0.001588251411  =============================================================     \|  \| **CervicalP14** \| **CervicalP7** \| **Cervical_P3** \| **LumbarP14** \| **LumbarP7** \| **Lumbar_P3** \| \| --- \| --- \| --- \| --- \| --- \| --- \| --- \| \| **CervicalP14** \| -1.000000 \| 0.804503 \| 0.943524 \| 0.869312 \| 0.892425 \| 0.246534 \| \| **CervicalP7** \| 0.804503 \| -1.000000 \| 0.869312 \| 0.191695 \| 0.925309 \| 0.925309 \| \| **Cervical_P3** \| 0.943524 \| 0.869312 \| -1.000000 \| 0.925309 \| 0.892425 \| 0.439295 \| \| **LumbarP14** \| 0.869312 \| 0.191695 \| 0.925309 \| -1.000000 \| 0.324884 \| 0.079463 \| \| **LumbarP7** \| 0.892425 \| 0.925309 \| 0.892425 \| 0.324884 \| -1.000000 \| 0.892425 \| \| **Lumbar_P3** \| 0.246534 \| 0.925309 \| 0.439295 \| 0.079463 \| 0.892425 \| -1.000000 \|     ------------------------------------------------------------- | mV | Cervical vs lumbar at ages groups P2-3,P6-7,P14-21 | Spearmans correlation coefficient  Mann Whitney U with Holm-Sidak correction | 1a |
|  |  |  |  |  |  |  |  |  |  |  |
| 2. Is development of whole cell capacitance (WCC) different in cervical and lumbar motoneurons | Significant increase with age in both segments. No difference between segments at any age. | Age: P2-3,P6-7,P14-21  Segment: Lumbar, Cervical  L P2-3  L P6-7 L P14-21 C P2-3  C P6-7  C P14-21 | 223  212  310  353  405  340 | 71  59  140  136  217  148 | 17  17  30  30  18  22 | Spearmans correlation coefficient: 0.343  Cervical Capacitance is correlated with age(reject H0) p=0.011883789311  Spearmans correlation coefficient: 0.502  lumbar Capacitance is correlated with age(reject H0) p=0.000109357489 ==================================================================   \|  \| **CervicalP14** \| **CervicalP7** \| **Cervical_P3** \| **LumbarP14** \| **LumbarP7** \| **Lumbar_P3** \| \| --- \| --- \| --- \| --- \| --- \| --- \| --- \| \| **CervicalP14** \| -1.000000 \| 0.944161 \| 0.015742 \| 0.944161 \| 0.944161 \| 0.027717 \| \| **CervicalP7** \| 0.944161 \| -1.000000 \| 0.004411 \| 0.944161 \| 0.769583 \| 0.006271 \| \| **Cervical_P3** \| 0.015742 \| 0.004411 \| -1.000000 \| 0.009281 \| 0.036652 \| 0.944161 \| \| **LumbarP14** \| 0.944161 \| 0.944161 \| 0.009281 \| -1.000000 \| 0.712333 \| 0.009646 \| \| **LumbarP7** \| 0.944161 \| 0.769583 \| 0.036652 \| 0.712333 \| -1.000000 \| 0.036652 \| \| **Lumbar_P3** \| 0.027717 \| 0.006271 \| 0.944161 \| 0.009646 \| 0.036652 \| -1.000000 \| | pF | Cervical vs lumbar at ages groups P2-3,P6-7,P14-21 | Spearmans correlation coefficient  Mann Whitney U with Holm-Sidak correction | 1b |
| 3. Is development of cell input resistance (IR) different in cervical and lumbar motoneurons | Sig effect of age in both segments. No difference between segments at any age | Age: P2-3,P6-7,P14-21  Segment: Lumbar, Cervical  L P2-3  L P6-7 L P14-21 C P2-3  C P6-7  C P14-21 | 79  52  31  74  43  25 | 47  32  23  31  34  7.5 | 17  17  30  30  18  22 | Spearmans correlation coefficient: -0.812  Cervical Resistance is correlated with age(reject H0) p=0.0000000000002  Spearmans correlation coefficient: -0.551  lumbar Resistance is correlated with age(reject H0) p=0.000015603994  ================================================================     \| **Resistance** \| **CervicalP14** \| **CervicalP7** \| **Cervical_P3** \| **LumbarP14** \| **LumbarP7** \| **Lumbar_P3** \| \| --- \| --- \| --- \| --- \| --- \| --- \| --- \| \| **CervicalP14** \| -1.000000 \| 0.011485 \| 0.000002 \| 0.958457 \| 0.000449 \| 0.000019 \| \| **CervicalP7** \| 0.011485 \| -1.000000 \| 0.000238 \| 0.181554 \| 0.181554 \| 0.002231 \| \| **Cervical_P3** \| 0.000002 \| 0.000238 \| -1.000000 \| 0.000357 \| 0.027939 \| 0.958457 \| \| **LumbarP14** \| 0.958457 \| 0.181554 \| 0.000357 \| -1.000000 \| 0.027939 \| 0.001377 \| \| **LumbarP7** \| 0.000449 \| 0.181554 \| 0.027939 \| 0.027939 \| -1.000000 \| 0.130577 \| \| **Lumbar_P3** \| 0.000019 \| 0.002231 \| 0.958457 \| 0.001377 \| 0.130577 \| -1.000000 \| | mΩ | Cervical vs lumbar at ages groups P2-3,P6-7,P14-21 | Spearmans correlation coefficient  Mann Whitney U with Holm-Sidak correction | 1c |
| 4. Is development of whole cell input Tau different in cervical and lumbar motoneurons | Sig effect of age in both segments. No difference between segment at any age. | Age: P2-3,P6-7,P14-21  Segment: Lumbar, Cervical  L P2-3  L P6-7 L P14-21 C P2-3  C P6-7  C P14-21 | 16  13  9.6  14  12  8.2 | 6.7  4.9  3.9  4.7  5.9  3.3 | 17  17  30  30  18  22 | Spearmans correlation coefficient: -0.663  Cervical Tau is correlated with age(reject H0) p=0.00000006  Spearmans correlation coefficient: -0.407  lumbar Tau is correlated with age(reject H0) p=0.002245806264  ==============================================================   \|  \| **CervicalP14** \| **CervicalP7** \| **Cervical_P3** \| **LumbarP14** \| **LumbarP7** \| **Lumbar_P3** \| \| --- \| --- \| --- \| --- \| --- \| --- \| --- \| \| **CervicalP14** \| -1.000000 \| 0.001823 \| 0.000597 \| 0.717378 \| 0.000923 \| 0.000566 \| \| **CervicalP7** \| 0.001823 \| -1.000000 \| 0.329702 \| 0.139555 \| 0.717378 \| 0.187319 \| \| **Cervical_P3** \| 0.000597 \| 0.329702 \| -1.000000 \| 0.024996 \| 0.717378 \| 0.717378 \| \| **LumbarP14** \| 0.717378 \| 0.139555 \| 0.024996 \| -1.000000 \| 0.069885 \| 0.015810 \| \| **LumbarP7** \| 0.000923 \| 0.717378 \| 0.717378 \| 0.069885 \| -1.000000 \| 0.662470 \| \| **Lumbar_P3** \| 0.000566 \| 0.187319 \| 0.717378 \| 0.015810 \| 0.662470 \| -1.000000 \|   --------------------------------------------------------------  -------------------------------------------------------------- | ms | Cervical vs lumbar at ages groups P2-3,P6-7,P14-21 | Spearmans correlation coefficient  Mann Whitney U with Holm-Sidak correction | 1d |
| 5. Is development of AP HW different in cervical and lumbar motoneurons | Sig effect of age in both segments.Cervical lower than lumbar at P7 | Age: P2-3,P6-7,P14-21  Segment: Lumbar, Cervical  L P2-3  L P6-7 L P14-21 C P2-3  C P6-7  C P14-21 | 1.2  1.0  0.8  1.1  0.8  0.6 | 0.2  0.2  0.2  0.2  0.2  0.1 | 17  17  30  30  18  22 | Spearmans correlation coefficient: -0.792  Cervical AP HW is correlated with age(reject H0) p=0.000000000002  Spearmans correlation coefficient: -0.620  lumbar AP HW is correlated with age(reject H0) p=0.000000568107  ==============================================================     \|  \| **CervicalP14** \| **CervicalP7** \| **Cervical_P3** \| **LumbarP14** \| **LumbarP7** \| **Lumbar_P3** \| \| --- \| --- \| --- \| --- \| --- \| --- \| --- \| \| **CervicalP14** \| -1.000000e+00 \| 0.001213 \| 0.000005 \| 0.151058 \| 3.113574e-08 \| 0.000002 \| \| **CervicalP7** \| 1.213485e-03 \| -1.000000 \| 0.009591 \| 0.340684 \| 7.773771e-03 \| 0.000360 \| \| **Cervical_P3** \| 4.809193e-06 \| 0.009591 \| -1.000000 \| 0.002409 \| 4.994970e-01 \| 0.340684 \| \| **LumbarP14** \| 1.510577e-01 \| 0.340684 \| 0.002409 \| -1.000000 \| 1.213485e-03 \| 0.000437 \| \| **LumbarP7** \| 3.113574e-08 \| 0.007774 \| 0.499497 \| 0.001213 \| -1.000000e+00 \| 0.015499 \| \| **Lumbar_P3** \| 1.788310e-06 \| 0.000360 \| 0.340684 \| 0.000437 \| 1.549902e-02 \| -1.000000 \|   -------------------------------------------------------------- | ms | Cervical vs lumbar at ages groups P2-3,P6-7,P14-21 | Spearmans correlation coefficient  Mann Whitney U with Holm-Sidak correction | 2a |
| 6. Is development of AP repolarisation rate different in cervical and lumbar motoneurons | Sig effect of age. Cervical higher at P7 and P14 | Age: P2-3,P6-7,P14-21  Segment: Lumbar, Cervical  L P2-3  L P6-7 L P14-21 C P2-3  C P6-7  C P14-21 | 65  80  115  77  100  149 | 13  15  34  22  28  35 | 17  17  30  30  18  22 | Spearmans correlation coefficient: 0.786  Cervical max rate depolarisation is correlated with age(reject H0) p=0.000000000003  Spearmans correlation coefficient: 0.630  lumbar max rate depolarisation is correlated with age(reject H0) p=0.000000331171  =================================================================     \|  \| **CervicalP14** \| **CervicalP7** \| **Cervical_P3** \| **LumbarP14** \| **LumbarP7** \| **Lumbar_P3** \| \| --- \| --- \| --- \| --- \| --- \| --- \| --- \| \| **CervicalP14** \| -1.000000e+00 \| 0.000078 \| 0.000004 \| 0.035205 \| 4.372097e-08 \| 0.000002 \| \| **CervicalP7** \| 7.774312e-05 \| -1.000000 \| 0.035205 \| 0.321199 \| 2.313149e-02 \| 0.000485 \| \| **Cervical_P3** \| 4.141059e-06 \| 0.035205 \| -1.000000 \| 0.011514 \| 5.798928e-01 \| 0.321199 \| \| **LumbarP14** \| 3.520527e-02 \| 0.321199 \| 0.011514 \| -1.000000 \| 2.988706e-03 \| 0.000283 \| \| **LumbarP7** \| 4.372097e-08 \| 0.023131 \| 0.579893 \| 0.002989 \| -1.000000e+00 \| 0.032303 \| \| **Lumbar_P3** \| 1.788310e-06 \| 0.000485 \| 0.321199 \| 0.000283 \| 3.230288e-02 \| -1.000000 \|   ----------------------------------------------------------------- | mV/ms-1 | Cervical vs lumbar at ages groups P2-3,P6-7,P14-21 | Spearmans correlation coefficient  Mann Whitney U with Holm-Sidak correction | 2b |
| 7. Is development of depolarisation rate different  In cervical and lumbar motoneurons | Sig effect of age in both segments. No difference between segments at any age | Age: P2-3,P6-7,P14-21  Segment: Lumbar, Cervical  L P2-3  L P6-7 L P14-21 C P2-3  C P6-7  C P14-21 | 152  166  212  150  192  255 | 33  34  53  51  44  46 | 17  17  30  30  18  22 | Spearmans correlation coefficient: 0.725  Cervical Max rate depolarisation is correlated with age(reject H0) p=0.000000000856  Spearmans correlation coefficient: 0.457  lumbar Max rate depolarisation is correlated with age(reject H0) p=0.000507842016  ==================================================================     \|  \| **CervicalP14** \| **CervicalP7** \| **Cervical_P3** \| **LumbarP14** \| **LumbarP7** \| **Lumbar_P3** \| \| --- \| --- \| --- \| --- \| --- \| --- \| --- \| \| **CervicalP14** \| -1.000000e+00 \| 0.000235 \| 0.000093 \| 0.110759 \| 7.360860e-07 \| 0.000004 \| \| **CervicalP7** \| 2.353316e-04 \| -1.000000 \| 0.032656 \| 0.425425 \| 7.105719e-02 \| 0.015934 \| \| **Cervical_P3** \| 9.300154e-05 \| 0.032656 \| -1.000000 \| 0.032609 \| 4.254248e-01 \| 0.558185 \| \| **LumbarP14** \| 1.107586e-01 \| 0.425425 \| 0.032609 \| -1.000000 \| 3.265568e-02 \| 0.010557 \| \| **LumbarP7** \| 7.360860e-07 \| 0.071057 \| 0.425425 \| 0.032656 \| -1.000000e+00 \| 0.443769 \| \| **Lumbar_P3** \| 3.837054e-06 \| 0.015934 \| 0.558185 \| 0.010557 \| 4.437687e-01 \| -1.000000 \|   ------------------------------------------------------------------ | mV/ms-1 | Cervical vs lumbar at ages groups P2-3,P6-7,P14-21 | Spearmans correlation coefficient  Mann Whitney U with Holm-Sidak correction | 2c |
| 8. Is development of AP amplitude different  In cervical and lumbar motoneurons | Sig effect of age in cervical mns only.  No difference between segments at any age. | Age: P2-3,P6-7,P14-21  Segment: Lumbar, Cervical  L P2-3  L P6-7 L P14-21 C P2-3  C P6-7  C P14-21 | 69  68  70  63  69  73 | 7.8  6.1  8.5  9.2  7.4  7.7 | 17  17  30  30  18  22 | Spearmans correlation coefficient: 0.375  Cervical AP amplitude is correlated with age(reject H0) p=0.005614887040  Spearmans correlation coefficient: -0.00016  lumbar AP amplitude is not correlated with age (fail to reject H0) p=0.99911  ===============================================================   \|  \| **CervicalP14** \| **CervicalP7** \| **Cervical_P3** \| **LumbarP14** \| **LumbarP7** \| **Lumbar_P3** \| \| --- \| --- \| --- \| --- \| --- \| --- \| --- \| \| **CervicalP14** \| -1.000000 \| 0.524742 \| 0.037702 \| 0.918330 \| 0.172242 \| 0.713561 \| \| **CervicalP7** \| 0.524742 \| -1.000000 \| 0.556003 \| 0.955139 \| 0.975123 \| 0.991167 \| \| **Cervical_P3** \| 0.037702 \| 0.556003 \| -1.000000 \| 0.379956 \| 0.712571 \| 0.644569 \| \| **LumbarP14** \| 0.918330 \| 0.955139 \| 0.379956 \| -1.000000 \| 0.892413 \| 0.975123 \| \| **LumbarP7** \| 0.172242 \| 0.975123 \| 0.712571 \| 0.892413 \| -1.000000 \| 0.975123 \| \| **Lumbar_P3** \| 0.713561 \| 0.991167 \| 0.644569 \| 0.975123 \| 0.975123 \| -1.000000 \|   --------------------------------------------------------------- | mV | Cervical vs lumbar at ages groups P2-3,P6-7,P14-21 | Spearmans correlation coefficient  Mann Whitney U with Holm-Sidak correction | 2d |
| 9. Is development of AP threshold different  In cervical and lumbar motoneurons | no effect of age | Age: P2-3,P6-7,P14-21  Segment: Lumbar, Cervical  L P2-3  L P6-7 L P14-21 C P2-3  C P6-7  C P14-21 | -36  -37  -35  -37  -34  -35 | 9.5  7.2  8.2  8.4  8.3  6.0 | 17  17  30  30  18  22 | Spearmans correlation coefficient: 0.148  Cervical AP thresh is not correlated with age (fail to reject H0) p=0.291  Spearmans correlation coefficient: 0.062  lumbar AP thresh is not correlated with age (fail to reject H0) p=0.656  ===============================================================  --------------------------------------------------------------- | mV | Cervical vs lumbar at ages groups P2-3,P6-7,P14-21 | Spearmans correlation coefficient  Mann Whitney U with Holm-Sidak correction | 2e |
| 10. Is development of AP fAHP amplitude different  In cervical and lumbar motoneurons | sig effect of age in both segments-sig difference between segments at P7 | Age: P2-3,P6-7,P14-21  Segment: Lumbar, Cervical  L P2-3  L P6-7 L P14-21 C P2-3  C P6-7  C P14-21 | -8.6  -9.9  -16  -11  -14  -19 | 7.5  3.5  3.6  4.1  4.8  5.5 | 17  17  30  30  18  22 | Spearmans correlation coefficient: -0.611  Cervical fAHP amplitude is correlated with age(reject H0) p=0.000001196202  Spearmans correlation coefficient: -0.511  lumbar fAHP amplitude is correlated with age(reject H0) p=0.000079188463  ==============================================================   \|  \| **CervicalP14** \| **CervicalP7** \| **Cervical_P3** \| **LumbarP14** \| **LumbarP7** \| **Lumbar_P3** \| \| --- \| --- \| --- \| --- \| --- \| --- \| --- \| \| **CervicalP14** \| -1.000000 \| 0.104510 \| 0.000736 \| 0.665259 \| 0.000002 \| 0.000204 \| \| **CervicalP7** \| 0.104510 \| -1.000000 \| 0.104510 \| 0.665259 \| 0.001974 \| 0.010969 \| \| **Cervical_P3** \| 0.000736 \| 0.104510 \| -1.000000 \| 0.009174 \| 0.665259 \| 0.665259 \| \| **LumbarP14** \| 0.665259 \| 0.665259 \| 0.009174 \| -1.000000 \| 0.000069 \| 0.001974 \| \| **LumbarP7** \| 0.000002 \| 0.001974 \| 0.665259 \| 0.000069 \| -1.000000 \| 0.665259 \| \| **Lumbar_P3** \| 0.000204 \| 0.010969 \| 0.665259 \| 0.001974 \| 0.665259 \| -1.000000 \|   -------------------------------------------------------------- | mV | Cervical vs lumbar at ages groups P2-3,P6-7,P14-21 | Spearmans correlation coefficient  Mann Whitney U with Holm-Sidak correction | 2f |
| 11. Is development of AP mAHP amplitude different  In cervical and lumbar motoneurons | sig effect of age in both segments-no difference between segments at any age | Age: P2-3,P6-7,P14-21  Segment: Lumbar, Cervical  L P2-3  L P6-7 L P14-21 C P2-3  C P6-7  C P14-21 | 5.3  4.1  3.0  5.4  4.4  3.3 | 2.4  1.9  1.6  1.9  2.6  1.3 | 17  17  20  19  18  22 | Spearmans correlation coefficient: -0.417  Cervical mAHP amplitude is correlated with age(reject H0) p=0.001870922263  Spearmans correlation coefficient: -0.382  lumbar mAHP amplitude is correlated with age(reject H0) p=0.004399552570  =============================================================   \|  \| **CervicalP14** \| **CervicalP7** \| **Cervical_P3** \| **LumbarP14** \| **LumbarP7** \| **Lumbar_P3** \| \| --- \| --- \| --- \| --- \| --- \| --- \| --- \| \| **CervicalP14** \| -1.000000 \| 0.278110 \| 0.002558 \| 0.989713 \| 0.549838 \| 0.043421 \| \| **CervicalP7** \| 0.278110 \| -1.000000 \| 0.525098 \| 0.278110 \| 0.885204 \| 0.566421 \| \| **Cervical_P3** \| 0.002558 \| 0.525098 \| -1.000000 \| 0.004659 \| 0.146385 \| 1.000000 \| \| **LumbarP14** \| 0.989713 \| 0.278110 \| 0.004659 \| -1.000000 \| 0.525098 \| 0.038940 \| \| **LumbarP7** \| 0.549838 \| 0.885204 \| 0.146385 \| 0.525098 \| -1.000000 \| 0.416870 \| \| **Lumbar_P3** \| 0.043421 \| 0.566421 \| 1.000000 \| 0.038940 \| 0.416870 \| -1.000000 \|   ------------------------------------------------------------- | mV | Cervical vs lumbar at ages groups P2-3,P6-7,P14-21 | Spearmans correlation coefficient  Mann Whitney U with Holm-Sidak correction | 3a |
| 12. Is development of AP mAHP decay different  In cervical and lumbar motoneurons | sig effect of age in cervical only. No difference between segments at any age. | Age: P2-3,P6-7,P14-21  Segment: Lumbar, Cervical  L P2-3  L P6-7 L P14-21 C P2-3  C P6-7  C P14-21 | 67  74  74  78  57  62 | 22  39  31  20  24  24 | 17  17  20  19  18  22 | Spearmans correlation coefficient: -0.398  Cervical mAHP 1/2 decay is correlated with age(reject H0) p=0.003144304736  Spearmans correlation coefficient: -0.005  lumbar mAHP 1/2 decay is not correlated with age (fail to reject H0) p=0.970  ===============================================================    ---------------------------------------------------------------   \| **mAHP decay** \| **CervicalP14** \| **CervicalP7** \| **Cervical_P3** \| **LumbarP14** \| **LumbarP7** \| **Lumbar_P3** \| \| --- \| --- \| --- \| --- \| --- \| --- \| --- \| \| **CervicalP14** \| -1.000000 \| 0.918921 \| 0.028786 \| 0.864517 \| 0.918921 \| 0.855480 \| \| **CervicalP7** \| 0.918921 \| -1.000000 \| 0.006035 \| 0.543922 \| 0.855480 \| 0.543922 \| \| **Cervical_P3** \| 0.028786 \| 0.006035 \| -1.000000 \| 0.864517 \| 0.918921 \| 0.864517 \| \| **LumbarP14** \| 0.864517 \| 0.543922 \| 0.864517 \| -1.000000 \| 0.998671 \| 0.995611 \| \| **LumbarP7** \| 0.918921 \| 0.855480 \| 0.918921 \| 0.998671 \| -1.000000 \| 0.998671 \| \| **Lumbar_P3** \| 0.855480 \| 0.543922 \| 0.864517 \| 0.995611 \| 0.998671 \| -1.000000 \| | mV | Cervical vs lumbar at ages groups P2-3,P6-7,P14-21 | Spearmans correlation coefficient  Mann Whitney U with Holm-Sidak correction | 3b |
| 13. Is development of AP ADP amplitude different  In cervical and lumbar motoneurons | sig effect of age in both segments-  no difs between segments at any age | Age: P2-3,P6-7,P14-21  Segment: Lumbar, Cervical  L P2-3  L P6-7 L P14-21 C P2-3  C P6-7  C P14-21 | 0.5  0.7  3.0  0.3  1.2  3.4 | 0.8  0.7  1.6  0.5  0.9  1.3 | 17  17  20  19  18  22 | Spearmans correlation coefficient: 0.832  Cervical ADP is correlated with age(reject H0) p=0.00000000000001  Spearmans correlation coefficient: 0.623  lumbar ADP is correlated with age(reject H0) p=0.000000486196  ==============================================================     \| ADP amp \| **CervicalP14** \| **CervicalP7** \| **Cervical_P3** \| **LumbarP14** \| **LumbarP7** \| **Lumbar_P3** \| \| --- \| --- \| --- \| --- \| --- \| --- \| --- \| \| **CervicalP14** \| -1.000000 \| 0.000082 \| 0.000007 \| 0.580422 \| 0.000002 \| 0.000007 \| \| **CervicalP7** \| 0.000082 \| -1.000000 \| 0.004292 \| 0.009987 \| 0.164041 \| 0.026885 \| \| **Cervical_P3** \| 0.000007 \| 0.004292 \| -1.000000 \| 0.000038 \| 0.282098 \| 0.580422 \| \| **LumbarP14** \| 0.580422 \| 0.009987 \| 0.000038 \| -1.000000 \| 0.000117 \| 0.000125 \| \| **LumbarP7** \| 0.000002 \| 0.164041 \| 0.282098 \| 0.000117 \| -1.000000 \| 0.580422 \| \| **Lumbar_P3** \| 0.000007 \| 0.026885 \| 0.580422 \| 0.000125 \| 0.580422 \| -1.000000 \|   -------------------------------------------------------------- | mV | Cervical vs lumbar at ages groups P2-3,P6-7,P14-21 | Spearmans correlation coefficient  Mann Whitney U with Holm-Sidak correction | 3c |
| 14. Is development of sag slope different  In cervical and lumbar motoneurons | sig effect of age-no effect of segment or interaction. | Age: P2-3,P6-7,P14-21  Segment: Lumbar, Cervical  L P2-3  L P6-7 L P14-21 C P2-3  C P6-7  C P14-21 | 7.7  4.4  6.1  6.7  2.1  5.5 | 7.3  4.8  4.0  8.0  2.6  2.9 | 17  17  30  30  18  22 | Two way ANOVA   \| **sum_sq** \| **df** \| **F** \| **PR(>F)** \| \| --- \| --- \| --- \| --- \| \| **C(Neuraxis)** \| 36.991359 \| 1.0 \| 1.641187 \| 0.202553 \| \| **C(agegroup)** \| 358.555232 \| 2.0 \| 7.953969 \| 0.000562 \| \| **C(Neuraxis):C(agegroup)** \| 16.776624 \| 2.0 \| 0.372162 \| 0.690010 \| \| **Residual** \| 2794.884438 \| 124.0 \| NaN \| NaN \|   ==============================================================     \|  \| **CervicalP14** \| **CervicalP7** \| **Cervical_P3** \| **LumbarP14** \| **LumbarP7** \| **Lumbar_P3** \| \| --- \| --- \| --- \| --- \| --- \| --- \| --- \| \| **CervicalP14** \| -1.000000 \| 0.232429 \| 0.798743 \| 0.900000 \| 0.900000 \| 0.726078 \| \| **CervicalP7** \| 0.232429 \| -1.000000 \| 0.022999 \| 0.133591 \| 0.340497 \| 0.013547 \| \| **Cervical_P3** \| 0.798743 \| 0.022999 \| -1.000000 \| 0.900000 \| 0.717386 \| 0.900000 \| \| **LumbarP14** \| 0.900000 \| 0.133591 \| 0.900000 \| -1.000000 \| 0.900000 \| 0.900000 \| \| **LumbarP7** \| 0.900000 \| 0.340497 \| 0.717386 \| 0.900000 \| -1.000000 \| 0.643884 \| \| **Lumbar_P3** \| 0.726078 \| 0.013547 \| 0.900000 \| 0.900000 \| 0.643884 \| -1.000000 \|   -------------------------------------------------------------- | mV/nA | Cervical vs lumbar at ages groups P2-3,P6-7,P14-21 | 2-way ANOVA  Tukey posthoc | 4a |
| 15. Is development of I min different  In cervical and lumbar motoneurons | sig effect of age-no difference between segments at any age | Age: P2-3,P6-7,P14-21  Segment: Lumbar, Cervical  L P2-3  L P6-7 L P14-21 C P2-3  C P6-7  C P14-21 | 0.5  0.5  1.5  0.4  0.8  1.3 | 0.4  0.3  1.1  0.2  0.5  0.7 | 17  17  30  30  18  22 | Spearmans correlation coefficient: 0.708  Cervical Rheobase is correlated with age(reject H0) p=0.000000003  Spearmans correlation coefficient: 0.548  lumbar Rheobase is correlated with age(reject H0) p=0.000017767654  ==============================================================     \|  \| **CervicalP14** \| **CervicalP7** \| **Cervical_P3** \| **LumbarP14** \| **LumbarP7** \| **Lumbar_P3** \| \| --- \| --- \| --- \| --- \| --- \| --- \| --- \| \| **CervicalP14** \| -1.000000 \| 0.052209 \| 0.000011 \| 0.992500 \| 0.000011 \| 0.000407 \| \| **CervicalP7** \| 0.052209 \| -1.000000 \| 0.003680 \| 0.136832 \| 0.054109 \| 0.038630 \| \| **Cervical_P3** \| 0.000011 \| 0.003680 \| -1.000000 \| 0.000629 \| 0.160094 \| 0.992500 \| \| **LumbarP14** \| 0.992500 \| 0.136832 \| 0.000629 \| -1.000000 \| 0.002477 \| 0.002394 \| \| **LumbarP7** \| 0.000011 \| 0.054109 \| 0.160094 \| 0.002477 \| -1.000000 \| 0.352622 \| \| **Lumbar_P3** \| 0.000407 \| 0.038630 \| 0.992500 \| 0.002394 \| 0.352622 \| -1.000000 \|   -------------------------------------------------------------- | nA | Cervical vs lumbar at ages groups P2-3,P6-7,P14-21 | Spearmans correlation coefficient  Mann Whitney U with Holm-Sidak correction | 5a |
|  |  |  |  |  |  |  |  |  |  |  |
| 16. Is development of max spike output different  In cervical and lumbar motoneurons | sig effect of age in cervical only. Cervical greater than lumbar at P6-7 | Age: P2-3,P6-7,P14-21  Segment: Lumbar (L), Cervical (C)  L P2-3  L P6-7 L P14-21 C P2-3  C P6-7  C P14-21 | 23  23  30  25  32  38 | 4.2  6.5  9.4  5.8  8.8  9.8 | 17  17  30  30  18  22 | Spearmans correlation coefficient: 0.567  Cervical Max spikes is correlated with age(reject H0) p=0.000009628514  Spearmans correlation coefficient: 0.248  lumbar Max spikes is not correlated with age (fail to reject H0) p=0.070  ===============================================================     \| **Max spikes** \| **CervicalP14** \| **CervicalP7** \| **Cervical_P3** \| **LumbarP14** \| **LumbarP7** \| **Lumbar_P3** \| \| --- \| --- \| --- \| --- \| --- \| --- \| --- \| \| **CervicalP14** \| -1.000000 \| 0.171365 \| 0.000482 \| 0.110284 \| 0.000012 \| 0.000127 \| \| **CervicalP7** \| 0.171365 \| -1.000000 \| 0.082395 \| 0.758585 \| 0.003243 \| 0.013625 \| \| **Cervical_P3** \| 0.000482 \| 0.082395 \| -1.000000 \| 0.169020 \| 0.741737 \| 0.737251 \| \| **LumbarP14** \| 0.110284 \| 0.758585 \| 0.169020 \| -1.000000 \| 0.076708 \| 0.082395 \| \| **LumbarP7** \| 0.000012 \| 0.003243 \| 0.741737 \| 0.076708 \| -1.000000 \| 0.902816 \| \| **Lumbar_P3** \| 0.000127 \| 0.013625 \| 0.737251 \| 0.082395 \| 0.902816 \| -1.000000 \| | Hz | Cervical vs lumbar at ages groups P2-3,P6-7,P14-21 | Spearmans correlation coefficient  Mann Whitney U with Holm-Sidak correction | 5b |
| 17. Is development of max initial frequency different  In cervical and lumbar motoneurons | sig effect of age in both segments-cervical higher than lumbar at P6 and P14 | Age: P2-3,P6-7,P14-21  Segment: Lumbar, Cervical  L P2-3  L P6-7 L P14-21 C P2-3  C P6-7  C P14-21 | 114  123  185  106  159  219 | 25  34  44  25  35  30 | 17  17  30  30  18  22 | Spearmans correlation coefficient: 0.841  Cervical Max initial frequency is correlated with age(reject H0) p=0.000000000000003  Spearmans correlation coefficient: 0.526  lumbar Max initial frequency is correlated with age(reject H0) p=0.000044111334  ==================================================================     \| **Max initial**  **freq** \| **CervicalP14** \| **CervicalP7** \| **Cervical_P3** \| **LumbarP14** \| **LumbarP7** \| **Lumbar_P3** \| \| --- \| --- \| --- \| --- \| --- \| --- \| --- \| \| **CervicalP14** \| -1.000000e+00 \| 3.857483e-07 \| 9.825474e-14 \| 0.025844 \| 4.087841e-13 \| 7.476242e-13 \| \| **CervicalP7** \| 3.857483e-07 \| -1.000000e+00 \| 2.176828e-05 \| 0.107753 \| 1.625493e-03 \| 2.644950e-04 \| \| **Cervical_P3** \| 9.825474e-14 \| 2.176828e-05 \| -1.000000e+00 \| 0.000003 \| 1.860895e-01 \| 5.226380e-01 \| \| **LumbarP14** \| 2.584387e-02 \| 1.077534e-01 \| 2.725815e-06 \| -1.000000 \| 2.176828e-05 \| 1.716163e-05 \| \| **LumbarP7** \| 4.087841e-13 \| 1.625493e-03 \| 1.860895e-01 \| 0.000022 \| -1.000000e+00 \| 5.226380e-01 \| \| **Lumbar_P3** \| 7.476242e-13 \| 2.644950e-04 \| 5.226380e-01 \| 0.000017 \| 5.226380e-01 \| -1.000000e+00 \|   ------------------------------------------------------------------ | Hz | Cervical vs lumbar at ages groups P2-3,P6-7,P14-21 | Spearmans correlation coefficient  Mann Whitney U with Holm-Sidak correction | 5d |
| 18. Is development of max final frequency different  In cervical and lumbar motoneurons | sig effect of age in both segments-cervical higher than lumbar at P6 | Age: P2-3,P6-7,P14-21  Segment: Lumbar, Cervical  L P2-3  L P6-7 L P14-21 C P2-3  C P6-7  C P14-21 | 44  46  56  47  61  69 | 8.1  15  17  9.9  16  19 | 17  17  30  30  18  22 | Spearmans correlation coefficient: 0.528  Cervical max final freqis correlated with age(reject H0) p=0.000048178072  Spearmans correlation coefficient: 0.282  lumbar max final freq is correlated with age(reject H0) p=0.038556241248  ================================================================     \|  \| **CervicalP14** \| **CervicalP7** \| **Cervical_P3** \| **LumbarP14** \| **LumbarP7** \| **Lumbar_P3** \| \| --- \| --- \| --- \| --- \| --- \| --- \| --- \| \| **CervicalP14** \| -1.000000 \| 0.518426 \| 0.002029 \| 0.243778 \| 0.000214 \| 0.000182 \| \| **CervicalP7** \| 0.518426 \| -1.000000 \| 0.043529 \| 0.821529 \| 0.008896 \| 0.004949 \| \| **Cervical_P3** \| 0.002029 \| 0.043529 \| -1.000000 \| 0.243778 \| 0.821529 \| 0.716605 \| \| **LumbarP14** \| 0.243778 \| 0.821529 \| 0.243778 \| -1.000000 \| 0.155155 \| 0.062517 \| \| **LumbarP7** \| 0.000214 \| 0.008896 \| 0.821529 \| 0.155155 \| -1.000000 \| 0.821529 \| \| **Lumbar_P3** \| 0.000182 \| 0.004949 \| 0.716605 \| 0.062517 \| 0.821529 \| -1.000000 \|   ---------------------------------------------------------------- | Hz | Cervical vs lumbar at ages groups P2-3,P6-7,P14-21 | Spearmans correlation coefficient  Mann Whitney U with Holm-Sidak correction | 5e |
|  |  |  |  |  |  |  |  |  |  |  |
|  |  |  |  |  |  |  |  |  |  |  |
|  |  |  |  |  |  |  |  |  |  |  |
|  |  |  |  |  |  |  |  |  |  |  |
|  |  |  |  |  |  |  |  |  |  |  |
|  |  |  |  |  |  |  |  |  |  |  |
|  |  |  |  |  |  |  |  |  |  |  |
|  |  |  |  |  |  |  |  |  |  |  |
| 19. Is development Spike number f-I slope different  In cervical and lumbar motoneurons | sig effect of age in both segments-cervical lower than lumbar at P6 | Age: P2-3,P6-7,P14-21  Segment: Lumbar, Cervical  L P2-3  L P6-7 L P14-21 C P2-3  C P6-7  C P14-21 | 29  25  11  28  17  11 | 15  13  4.1  10  6.8  3.6 | 17  17  30  30  18  22 | Spearmans correlation coefficient: -0.706  Cervical spike slope is correlated with age(reject H0) p=0.000000003618  Spearmans correlation coefficient: -0.699  lumbar spike slope is correlated with age(reject H0) p=0.000000004153  ================================================================     \| **Spike slope** \| **CervicalP14** \| **CervicalP7** \| **Cervical_P3** \| **LumbarP14** \| **LumbarP7** \| **Lumbar_P3** \| \| --- \| --- \| --- \| --- \| --- \| --- \| --- \| \| **CervicalP14** \| -1.000000e+00 \| 0.003185 \| 0.000008 \| 0.899673 \| 1.005716e-07 \| 0.000004 \| \| **CervicalP7** \| 3.185486e-03 \| -1.000000 \| 0.001951 \| 0.024395 \| 2.156706e-03 \| 0.001951 \| \| **Cervical_P3** \| 7.971248e-06 \| 0.001951 \| -1.000000 \| 0.000017 \| 6.825863e-01 \| 0.958783 \| \| **LumbarP14** \| 8.996727e-01 \| 0.024395 \| 0.000017 \| -1.000000 \| 1.501056e-06 \| 0.000017 \| \| **LumbarP7** \| 1.005716e-07 \| 0.002157 \| 0.682586 \| 0.000002 \| -1.000000e+00 \| 0.749637 \| \| **Lumbar_P3** \| 4.124071e-06 \| 0.001951 \| 0.958783 \| 0.000017 \| 7.496370e-01 \| -1.000000 \|   ------------------------------------------------------------------------------------------------------------------------------- | Hz/nA | Cervical vs lumbar at ages groups P2-3,P6-7,P14-21 | Spearmans correlation coefficient  Mann Whitney U with Holm-Sidak correction | 6a |
| 20. Is development Initial frequency gain different  In cervical and lumbar motoneurons | sig effect of age in both segments-cervical lower than lumbar at P6 | Age: P2-3,P6-7,P14-21  Segment: Lumbar, Cervical  L P2-3  L P6-7 L P14-21 C P2-3  C P6-7  C P14-21 | 149  139  72  137  86  75 | 84  101  30  53  37  29 | 17  17  30  30  18  22 | Spearmans correlation coefficient: -0.493  Cervical initial interval slope is correlated with age(reject H0) p=0.000177531936  Spearmans correlation coefficient: -0.550  lumbar initial interval slope is correlated with age(reject H0) p=0.000016805453  ================================================================     \|  \| **CervicalP14** \| **CervicalP7** \| **Cervical_P3** \| **LumbarP14** \| **LumbarP7** \| **Lumbar_P3** \| \| --- \| --- \| --- \| --- \| --- \| --- \| --- \| \| **CervicalP14** \| -1.000000 \| 0.900656 \| 0.001659 \| 0.900656 \| 0.003072 \| 0.003663 \| \| **CervicalP7** \| 0.900656 \| -1.000000 \| 0.003663 \| 0.852061 \| 0.015751 \| 0.008702 \| \| **Cervical_P3** \| 0.001659 \| 0.003663 \| -1.000000 \| 0.003663 \| 0.900656 \| 0.972523 \| \| **LumbarP14** \| 0.900656 \| 0.852061 \| 0.003663 \| -1.000000 \| 0.003663 \| 0.004470 \| \| **LumbarP7** \| 0.003072 \| 0.015751 \| 0.900656 \| 0.003663 \| -1.000000 \| 0.900656 \| \| **Lumbar_P3** \| 0.003663 \| 0.008702 \| 0.972523 \| 0.004470 \| 0.900656 \| -1.000000 \|   ---------------------------------------------------------------- | Hz/nA | Cervical vs lumbar at ages groups P2-3,P6-7,P14-21 | Spearmans correlation coefficient  Mann Whitney U with Holm-Sidak correction | 6b |
| 21. Is development final frequency gain different  In cervical and lumbar motoneurons | sig effect of age in both segments. Cervical greater than lumbar at P7 | Age: P2-3,P6-7,P14-21  Segment: Lumbar, Cervical  L P2-3  L P6-7 L P14-21 C P2-3  C P6-7  C P14-21 | 51  40  19  51  28  18 | 29  19  7.9  16  12  5.1 | 17  17  30  30  18  22 | Spearmans correlation coefficient: -0.712  Cervical SS_slope is correlated with age(reject H0) p=0.000000002280  Spearmans correlation coefficient: -0.681  lumbar SS_slope is correlated with age(reject H0) p=0.000000014579  ================================================================     \|  \| **CervicalP14** \| **CervicalP7** \| **Cervical_P3** \| **LumbarP14** \| **LumbarP7** \| **Lumbar_P3** \| \| --- \| --- \| --- \| --- \| --- \| --- \| --- \| \| **CervicalP14** \| -1.000000e+00 \| 0.004729 \| 0.000002 \| 0.740822 \| 9.558837e-08 \| 0.000003 \| \| **CervicalP7** \| 4.729335e-03 \| -1.000000 \| 0.000182 \| 0.048454 \| 1.165733e-02 \| 0.001818 \| \| **Cervical_P3** \| 1.788310e-06 \| 0.000182 \| -1.000000 \| 0.000014 \| 3.520527e-02 \| 0.740822 \| \| **LumbarP14** \| 7.408220e-01 \| 0.048454 \| 0.000014 \| -1.000000 \| 1.724922e-05 \| 0.000041 \| \| **LumbarP7** \| 9.558837e-08 \| 0.011657 \| 0.035205 \| 0.000017 \| -1.000000e+00 \| 0.315125 \| \| **Lumbar_P3** \| 3.063216e-06 \| 0.001818 \| 0.740822 \| 0.000041 \| 3.151249e-01 \| -1.000000 \|   ---------------------------------------------------------------- | Hz | Cervical vs lumbar at ages groups P2-3,P6-7,P14-21 | Spearmans correlation coefficient  Mann Whitney U with Holm-Sidak correction | 6c |
| 22. Does age influence PC1 and is there a difference between lumbar and cervical motoneurons? | Significant change with development for both segements and earlier development in cervical | Age: P2-3,P6-7,P14-21  Segment: Lumbar, Cervical  L P2-3  L P6-7 L P14-21 C P2-3  C P6-7  C P14-21 | 2.40  2.93  -1.76  2.40  -1.32  -3.15 | 2.03  1.21  2.47  1.77  1.47  1.13 | 17  20  17  15  18  22 | Spearmans correlation coefficient: -0.797  Cervical PC1 interval slope is correlated with age(reject H0) p=0.000000000001  Spearmans correlation coefficient: -0.608  lumbar PC1 interval slope is correlated with age(reject H0) p=0.000002781086   \| PC1 \| **CervicalP14** \| **CervicalP7** \| **Cervical_P3** \| **LumbarP14** \| **LumbarP7** \| **Lumbar_P3** \| \| --- \| --- \| --- \| --- \| --- \| --- \| --- \| \| **CervicalP14** \| -1.000000e+00 \| 7.297665e-04 \| 8.988810e-12 \| 0.168580 \| 1.665335e-15 \| 3.030909e-12 \| \| **CervicalP7** \| 7.297665e-04 \| -1.000000e+00 \| 2.288249e-06 \| 0.824607 \| 2.861775e-08 \| 4.217833e-07 \| \| **Cervical_P3** \| 8.988810e-12 \| 2.288249e-06 \| -1.000000e+00 \| 0.000147 \| 8.246073e-01 \| 8.246073e-01 \| \| **LumbarP14** \| 1.685799e-01 \| 8.246073e-01 \| 1.470421e-04 \| -1.000000 \| 1.026950e-05 \| 3.215247e-05 \| \| **LumbarP7** \| 1.665335e-15 \| 2.861775e-08 \| 8.246073e-01 \| 0.000010 \| -1.000000e+00 \| 4.497549e-01 \| \| **Lumbar_P3** \| 3.030909e-12 \| 4.217833e-07 \| 8.246073e-01 \| 0.000032 \| 4.497549e-01 \| -1.000000e+00 \| |  | Cervical vs lumbar at ages groups P2-3,P6-7,P14-21 | Spearmans correlation coefficient  T tests with Holm-Sidak correction | 7a |
| 23. Does age influence PC2 and is there a difference between lumbar and cervical motoneurons? | No change | Age: P2-3,P6-7,P14-21  Segment: Lumbar, Cervical  L P2-3  L P6-7 L P14-21 C P2-3  C P6-7  C P14-21 | 0.17  -0.42  0.18  0.32  -0.68  0.47 | 1.59  1.69  1.65  1.76  0.96  1.19 | 17  20  17  15  18  22 | Spearmans correlation coefficient: 0.111  Cervical PC2 is not correlated with age (fail to reject H0) p=0.430  Spearmans correlation coefficient: -0.010  lumbar PC2 slope is not correlated with age (fail to reject H0) p=0.947 |  | Cervical vs lumbar at ages groups P2-3,P6-7,P14-21 | Spearmans correlation coefficient  Mann Whitney U with Holm-Sidak correction | 7d |
| 24. Does age influence PC3 and is there a difference between lumbar and cervical motoneurons? | Significant change with development in cervical only. no difference between segments at any age | Age: P2-3,P6-7,P14-21  Segment: Lumbar, Cervical  L P2-3  L P6-7 L P14-21 C P2-3  C P6-7  C P14-21 | 0.08  -0.16  0.12  -0.21  -0.33  0.45 | 2.29  1.55  0.90  1.02  1.03  0.53 | 17  20  17  15  18  22 | Spearmans correlation coefficient: 0.307  Cervical PC3 interval slope is correlated with age(reject H0) p=0.025469187275  Spearmans correlation coefficient: 0.146  lumbar PC3 slope is not correlated with age (fail to reject H0) p=0.311   \| PC3 \| **CervicalP14** \| **CervicalP7** \| **Cervical_P3** \| **LumbarP14** \| **LumbarP7** \| **Lumbar_P3** \| \| --- \| --- \| --- \| --- \| --- \| --- \| --- \| \| **CervicalP14** \| -1.000000 \| 0.037549 \| 0.545985 \| 0.480267 \| 0.016609 \| 0.457201 \| \| **CervicalP7** \| 0.037549 \| -1.000000 \| 0.997812 \| 0.997812 \| 0.997812 \| 0.997812 \| \| **Cervical_P3** \| 0.545985 \| 0.997812 \| -1.000000 \| 0.997812 \| 0.997812 \| 0.997812 \| \| **LumbarP14** \| 0.480267 \| 0.997812 \| 0.997812 \| -1.000000 \| 0.951504 \| 0.997062 \| \| **LumbarP7** \| 0.016609 \| 0.997812 \| 0.997812 \| 0.951504 \| -1.000000 \| 0.997812 \| \| **Lumbar_P3** \| 0.457201 \| 0.997812 \| 0.997812 \| 0.997062 \| 0.997812 \| -1.000000 \| |  | Cervical vs lumbar at ages groups P2-3,P6-7,P14-21 | Spearmans correlation coefficient  Mann Whitney U with Holm-Sidak correction | 7e |
| 25. Does age influence PC4 and is there a difference between lumbar and cervical motoneurons? | Significant change with development in both segments. No difference between segments at any age | Age: P2-3,P6-7,P14-21  Segment: Lumbar, Cervical  L P2-3  L P6-7 L P14-21 C P2-3  C P6-7  C P14-21 | -0.60  0.09  0.97  -0.49  -0.38  0.38 | 1.13  0.99  1.56  0.89  1.09  1.04 | 17  20  17  15  18  22 | Spearmans correlation coefficient: 0.321  Cervical PC4 interval slope is correlated with age(reject H0) p=0.018914558617  Spearmans correlation coefficient: 0.459  lumbar PC4 interval slope is correlated with age(reject H0) p=0.000811734028   \| **PC4** \| **CervicalP14** \| **CervicalP7** \| **Cervical_P3** \| **LumbarP14** \| **LumbarP7** \| **Lumbar_P3** \| \| --- \| --- \| --- \| --- \| --- \| --- \| --- \| \| **CervicalP14** \| -1.000000 \| 0.445342 \| 0.090724 \| 0.719424 \| 0.908558 \| 0.029281 \| \| **CervicalP7** \| 0.445342 \| -1.000000 \| 0.985577 \| 0.213331 \| 0.740388 \| 0.908558 \| \| **Cervical_P3** \| 0.090724 \| 0.985577 \| -1.000000 \| 0.162079 \| 0.513125 \| 0.908558 \| \| **LumbarP14** \| 0.719424 \| 0.213331 \| 0.162079 \| -1.000000 \| 0.525776 \| 0.068376 \| \| **LumbarP7** \| 0.908558 \| 0.740388 \| 0.513125 \| 0.525776 \| -1.000000 \| 0.169474 \| \| **Lumbar_P3** \| 0.029281 \| 0.908558 \| 0.908558 \| 0.068376 \| 0.169474 \| -1.000000 \| |  | Cervical vs lumbar at ages groups P2-3,P6-7,P14-21 | Spearmans correlation coefficient  Mann Whitney U with Holm-Sidak correction | 7f |
| 26. Does age influence PC5 and is there a difference between lumbar and cervical motoneurons? | Significant change with development for both segments. Differences between segments P6-7 and P14-21 | Age: P2-3,P6-7,P14-21  Segment: Lumbar, Cervical  L P2-3  L P6-7 L P14-21 C P2-3  C P6-7  C P14-21 | 0.18  0.47  0.97  -0.49  -0.38  0.38 | 1.18  0.74  1.04  0.88  0.71  0.81 | 17  20  17  15  18  22 | Spearmans correlation coefficient: 0.375  Cervical PC5 interval slope is correlated with age(reject H0) p=0.005623144088  Spearmans correlation coefficient: -0.329  lumbar PC5 interval slope is correlated with age(reject H0) p=0.019777941188   \| PC5 \| **CervicalP14** \| **CervicalP7** \| **Cervical_P3** \| **LumbarP14** \| **LumbarP7** \| **Lumbar_P3** \| \| --- \| --- \| --- \| --- \| --- \| --- \| --- \| \| **CervicalP14** \| -1.000000 \| 0.115100 \| 0.115100 \| 0.025935 \| 0.936333 \| 0.936333 \| \| **CervicalP7** \| 0.115100 \| -1.000000 \| 0.936333 \| 0.711463 \| 0.024417 \| 0.606833 \| \| **Cervical_P3** \| 0.115100 \| 0.936333 \| -1.000000 \| 0.910091 \| 0.029700 \| 0.601947 \| \| **LumbarP14** \| 0.025935 \| 0.711463 \| 0.910091 \| -1.000000 \| 0.006397 \| 0.248730 \| \| **LumbarP7** \| 0.936333 \| 0.024417 \| 0.029700 \| 0.006397 \| -1.000000 \| 0.910091 \| \| **Lumbar_P3** \| 0.936333 \| 0.606833 \| 0.601947 \| 0.248730 \| 0.910091 \| -1.000000 \| |  | Cervical vs lumbar at ages groups P2-3,P6-7,P14-21 | Spearmans correlation coefficient  Mann Whitney U with Holm-Sidak correction | 7g |

*You may use multiple lines for the same question to indicate multiple comparisons

** Authors may wish to make the text bold where p is considered significant against a stated confidence limit
